# Bile acid retention in efferocytic macrophages shapes their inflammatory status during cholangitis

**DOI:** 10.1084/jem.20242079

**Published:** 2026-06-23

**Authors:** Amirah Al Jawazneh, Imke Liebold, Stephanie Leyk, Clarissa Lanzloth, Vera Brackrock, Tamara López-López, Sebastian Graute, Irene Aranda-Pardos, Madeleine Hamley, Jonas Bahn, Joerg Heeren, Johann Von Felden, Ansgar W. Lohse, Christoph Schramm, Dorothee Schwinge, Pablo J. Sáez, Noelia A-Gonzalez, Samuel Huber, Thomas Jacobs, Lorenz Adlung, Anna Worthmann, Lidia Bosurgi

**Affiliations:** 1 I. Department of Medicine, University Medical Center Hamburg-Eppendorf, Hamburg, Germany; 2 Protozoa Immunology, Bernhard Nocht Institute for Tropical Medicine, Hamburg, Germany; 3 Department of Biochemistry and Molecular Cell Biology, Center for Experimental Medicine, University Medical Center Hamburg-Eppendorf, Hamburg, Germany; 4 Institute of Immunology, University of Münster, Münster, Germany; 5 Hamburg Center for Translational Immunology, University Medical Center Hamburg-Eppendorf, Hamburg, Germany; 6 European Reference Network on Hepatological Diseases (ERN-RARE LIVER), Hamburg, Germany; 7 Center for Biomedical AI, University Medical Center Hamburg-Eppendorf, Hamburg, Germany

## Abstract

The clearance of apoptotic cells by phagocytes is crucial for restoring tissue balance after injury. In autoimmune liver diseases like primary sclerosing cholangitis, cell death is thought to result from accumulation of toxic bile acids within parenchymal cells. Whether, in this context, bile acid–loaded dying cells impact the efficiency of phagocytic macrophages in restoring tissue balance remains unknown. Here, we demonstrate that in a murine model of cholangitis, bile acids accumulate in a subpopulation of efferocytic macrophages with pro-inflammatory features. Our in vitro results indicate that, upon their engulfment, apoptotic hepatocytes laden with bile acids can serve as Trojan horses, delivering bile acids into efferocytic macrophages and thereby shaping macrophage function. This contrasts with the characteristics of macrophages that engulf apoptotic parenchymal cells lacking bile acids. Together, our findings delineate a system in which the content of the phagocytosed dying cells, specifically bile acid–laden hepatocytes, drives a pro-inflammatory program in the corresponding efferocytic macrophages, potentially contributing to chronic hepatic inflammation.

## Teaser

Al Jawazneh, Liebold, Leyk et al. show that engulfment of bile acid–loaded hepatic parenchymal cells results in the transfer of bile acids to phagocytic macrophages, altering their inflammatory profile. This identifies dying cells, and specifically their bile acid content, as active modulators of macrophage function, potentially driving chronic inflammation in cholangiopathies such as primary sclerosing cholangitis.

## Graphical Abstract

**Figure:**
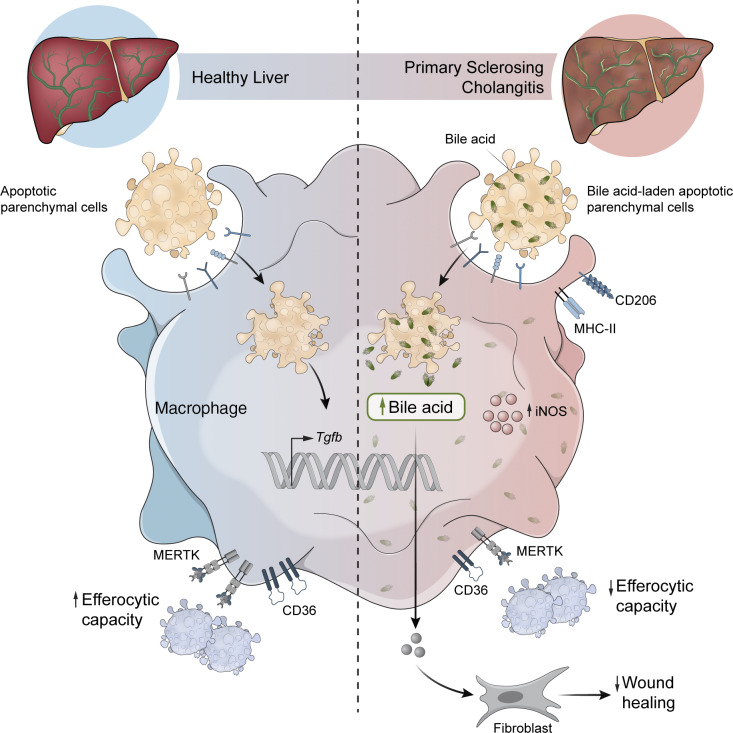

## Introduction

Macrophages (Mφ) are equipped to respond to a large array of stimuli to secure the acquisition of a proper activation status and therefore the generation of an appropriate immune response. In each tissue of our body, Mφ sense “universal stimuli” such as dying cells generated upon tissue damage, and thereby acquire “universal functions” as the capacity to engulf these cells through a process known as efferocytosis.

Proper efferocytosis guarantees maintenance of homeostasis, hence preventing the accumulation of dead cells and debris, which might be a relevant source of autoantigens. Indeed, failure in the process of dead cell clearance has been strongly associated with the development of several autoimmune diseases such as systemic lupus erythematosus, rheumatoid arthritis, and type 1 diabetes (Xing et al., 2024; Green et al., 2016; Martinez, 2017). Additionally, reduced phagocytic capacity has been observed in peripheral blood mononuclear cells (PBMCs) isolated from patients with autoimmune hepatitis (Lin et al., 2016).

In contrast to the universal functions exerted by Mφ all over the body, in certain tissues and in particular inflammatory settings, Mφ acquire “specialized functions” in response to tissue-specific signals (Gordon and Martinez, 2010; Gordon and Pluddemann, 2017; Okabe, 2018).

Bile acids represent a class of tissue-specific mediators, whose effects on Mφ activation status have been scarcely investigated to date. Primary bile acids are exclusively synthetized in the liver, as a result of cholesterol catabolism in the hepatocytes (Hofmann, 2009; Russell and Setchell, 1992), with around 5% of them being modified in the intestinal lumen, leading to the formation of secondary bile acids (Fleishman and Kumar, 2024).

Cholestatic liver diseases such as primary biliary cholangitis (PBC) and primary sclerosing cholangitis (PSC) are autoimmune diseases characterized by impaired bile excretion, which causes accumulation of bile acids (Karlsen et al., 2017; Tanaka et al., 2024). In contrast to the more hydrophilic ones, hydrophobic bile acids tend to be toxic and have been demonstrated to trigger hepatocyte cytotoxicity both in vitro and in vivo experimental settings (Sokol et al., 1995; Sokol et al., 1998; Spivey et al., 1993; Galle et al., 1990; Faubion et al., 1999; Attili et al., 1986).

Based on these findings, the retention of toxic bile acids in the liver has been suggested to contribute to hepatocyte and cholangiocyte damage and cell death (Guicciardi and Gores, 2002; Cai and Boyer, 2021; Li et al., 2024), with apoptosis representing a key regulator of cholestatic liver injury (Takeda et al., 2008; Miyoshi et al., 1999). Nevertheless, the role of bile acids in driving the development of cholestatic liver injury is still not fully understood.

When recurrent damage occurs, chronic cholestasis can develop into fibrosis, cirrhosis, and even liver failure. Here, reducing Mφ infiltration to the damaged liver via treatment with the dual CCR2/CCR5 antagonist (Guicciardi et al., 2018), via myeloid-specific deletion of *Arid3a* (Chen et al., 2023), or via IFNγ depletion (Ravichandran et al., 2019) diminishes periportal fibrosis, thus suggesting that Mφ accumulation in cholestatic mouse models is more harmful than helpful (Trussoni and LaRusso, 2023). In line with this, recently published data show Mφ-expressing *ETS2*, a central regulator of their inflammatory response, localizing in close proximity to cholangiocytes in liver biopsies from people with PSC, as detected via spatial transcriptomics (Stankey et al., 2024). Interestingly, in those pathological settings, hepatic Mφ are not only exposed to an increased amount of dying parenchymal cells, which need to be promptly cleared to prevent chronic liver inflammation but also to tissue-specific signals, such as toxic bile acids, which accumulate in the damaged liver.

Bile acids, per se, function as biological detergents that enable emulsification, digestion, and intestinal absorption of lipids and fat-soluble vitamins and have been used therapeutically for the treatment of various disorders (Staels and Fonseca, 2009). Ursodeoxycholic acid, for instance, is the drug of choice for the treatment of cholestatic liver diseases such as PBC and PSC (Camilleri and Gores, 2015). Similarly, in a mouse model of intestinal inflammation, administration of either primary or secondary bile acids has been reported to alleviate disease progression via modulating the frequency and function of T helper 17 (Th17)- regulatory T (Treg) cells (Song et al., 2020; Hang et al., 2019). Treatment in vitro of human and mouse T cells with lithocholic acid (LCA) inhibits Th1 activation (Pols et al., 2017). A similar immunosuppressive/anti-inflammatory profile has been detected in LPS-activated human Mφ treated in vitro with bile acids. Taurolithocholic acid (TLCA) stimulation in this case leads to reduced expression of the pro-inflammatory cytokine transcripts *IL6*, *TNF*, and *IL1* and increased expression of metalloprotease genes such as *MMP10* and *MMP12*, which are associated with a Mφ tissue remodeling function (Wammers et al., 2018). Similarly, LPS-activated rabbit alveolar Mφ show reduced expression of *Tnfa*, *Il1a*, *Il1b*, *Il6*, and *Il8* upon stimulation with TLCA and reduced capacity to uptake yeast cells in vitro (Kawamata et al., 2003). Additionally, conditioned media from Mφ treated in vitro with TLCA leads to biliary epithelial cell proliferation via induction of ITGβ6, hence further highlighting bile acids as a trigger of a tissue remodeling function in Mφ (Guillot et al., 2021; Hogenauer et al., 2014; Biagioli et al., 2017). However, despite their reported anti-inflammatory properties on both innate and adaptive immune response, the relevance of bile acids as therapeutic option still remains elusive, and data reporting detrimental effects of bile acids, particularly secondary bile acids, in the context of tumor growth, have also been reported (Ma et al., 2018).

We have recently described that dying cells with different cellular identities, once phagocytosed, drive transcriptional and functional heterogeneity in the corresponding phagocytic Mφ (Liebold et al., 2024). This emphasizes the fact that the content of the dying cells phagocytosed might contribute to the functional diversification observed in the targeted efferocytic Mφ. In this study, we found that in vitro–dying hepatocytes function as Trojan horses, carrying bile acids into a population of efferocytic Mφ. Contrary to what is known so far, this alternative mechanism of bile acid sensing promotes the acquisition of a pro-inflammatory phenotype in Mφ, associated with reduced efferocytic capacity and dysregulation of fibroblast wound healing function.

This previously unrecognized strategy of sensing bile acids represents a novel pathogenic process by which Mφ might sustain inflammation and aggravates liver disease. All in all, our work provides a framework in which interaction among apoptotic parenchymal cells, bile acids, and efferocytic Mφ represents a novel targetable process for prevention of liver disease progression and chronicity.

## Results

### Bile acids accumulate in liver efferocytic Mφ during cholangitis

**Figure 1.**
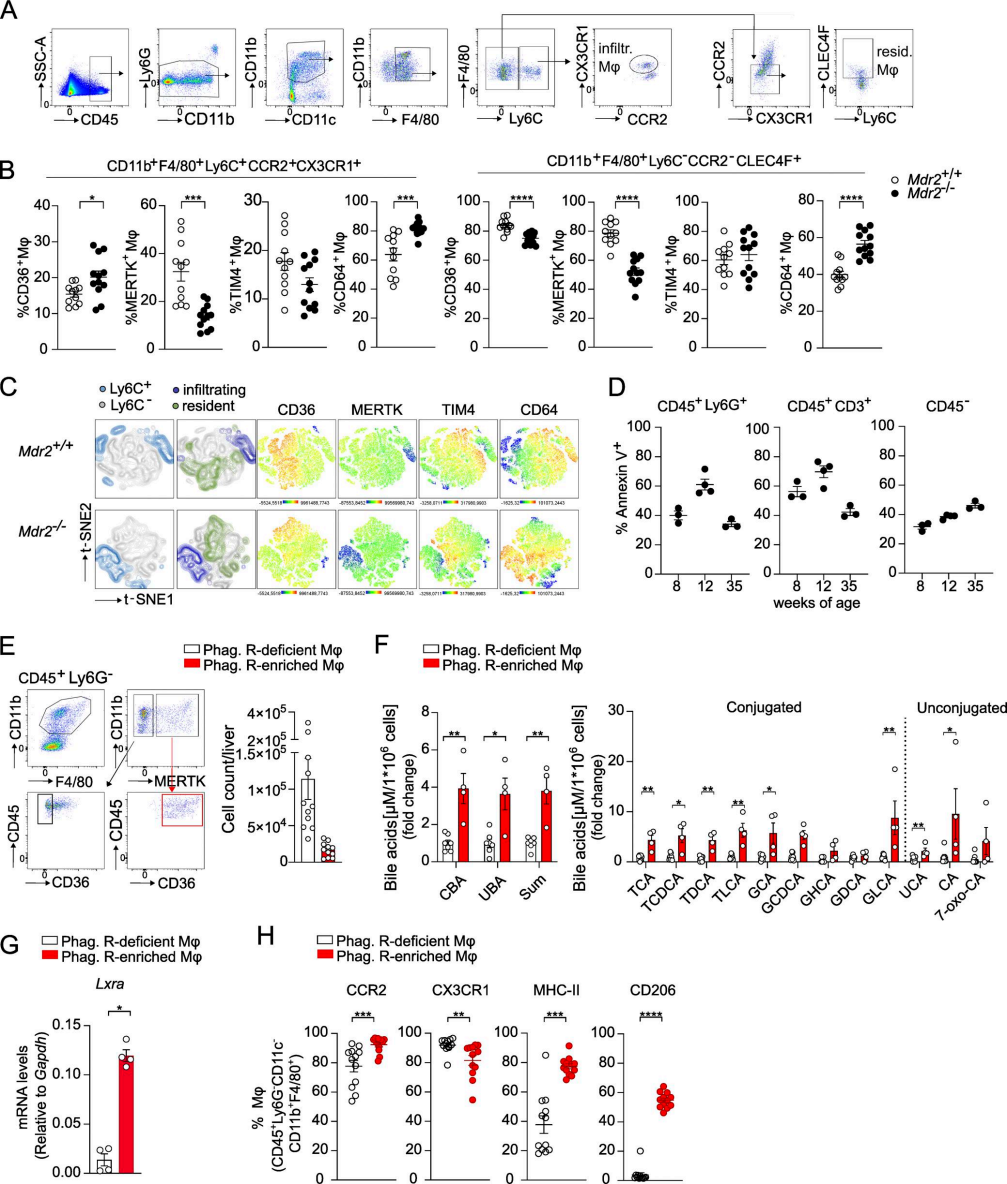
**Hepatic Mφ** **in *Mdr2*** ^**−/−**^ **mice show an altered efferocytic signature and accumulation of bile acids.** **(A)** Flow cytograms representing the gating strategy adopted for analysis of hepatic Mφ. Dead cells and doublets were excluded. Mφ were gated as CD45^+^Ly6G^−^CD11c^dim/lo^CD11b^+^F4/80^+^ and further divided into infiltrating (Ly6C^+^CCR2^+^CX3CR1^+^) and resident (Ly6C^−^CCR2^−^CX3CR1^−^CLEC4F^+^) Mφ. **(B)** Pooled data reporting frequency of infiltrating and resident Mφ expressing the receptors CD36, MERTK, TIM4, and CD64 isolated from the liver of *Mdr2*^*+/+*^ (clear dots) and *Mdr2*^*−/−*^ (filled dots) mice at 12 wk of age. *n* = 11–12 mice/time point. Mean ± SEM. Mann–Whitney U test. **(C)** t-SNE maps, concatenated from 11 to 12 mice/condition, showing the expression of the receptors CD36, MERTK, TIM4, and CD64 in infiltrating and resident Mφ within the Ly6C^+^ and Ly6C^−^ clusters in 12-wk-old *Mdr2*^*+/+*^ and *Mdr2*^*−/−*^ mice. **(D)** Percentage of Annexin V^+^ cells within neutrophils (CD45^+^Ly6G^+^), T cells (CD45^+^CD3^+^), and parenchymal cells (CD45^−^) isolated from the liver of *Mdr2*^*−/−*^ mice at 8, 12, and 35 wk of age and analyzed by flow cytometry. *n* = 3–4 mice/time point. **(E)** Flow cytograms depicting gating strategy for FACS-sorting of hepatic Mφ (CD45^+^Ly6G^−^ CD11b^+^F4/80^+^) isolated from 8- to 12-wk-old *Mdr2*^−/−^ mice, based on the expression of the efferocytic receptors MERTK and CD36 (as Phag. R–deficient Mφ or Phag. R–enriched Mφ). A bar graph reporting their absolute number per liver is also shown. **(F)** Quantification of conjugated bile acids (CBAs), unconjugated bile acids (UBAs), and the sum of all analyzed bile acids are classified based on bile acid classes. Data are presented as fold change relative to Phag. R–deficient Mφ. Four independent experiments were performed. Each dot corresponds to a pool of 3–6 mice. *n* = 4–7; mean ± SEM, Mann–Whitney U test. **(G)***Lxra* mRNA amount in Phag. R–enriched Mφ or Phag. R–deficient Mφ FACS-sorted as reported in E from 8- to 12-wk-old *Mdr2*^*−/−*^ mice, detected by qPCR. Four independent experiments were performed. *n* = 6–13 liver pooled/experiment; Mean ± SEM. Mann–Whitney U test. **(H)** Frequencies of Phag. R–enriched Mφ or Phag. R–deficient Mφ expressing CCR2, CX3CR1, MHC-II, and CD206 (out of CD45^+^Ly6G^−^CD11c^−^CD11b^+^F4/80^+^ cells), retrieved from the liver of *Mdr2*^*−/−*^ mice. *n* = 12 mice; mean ± SEM. Mann–Whitney U test. *P < 0.05; **P < 0.01; ***P < 0.001; ****P < 0.0001.

**Figure S1.**
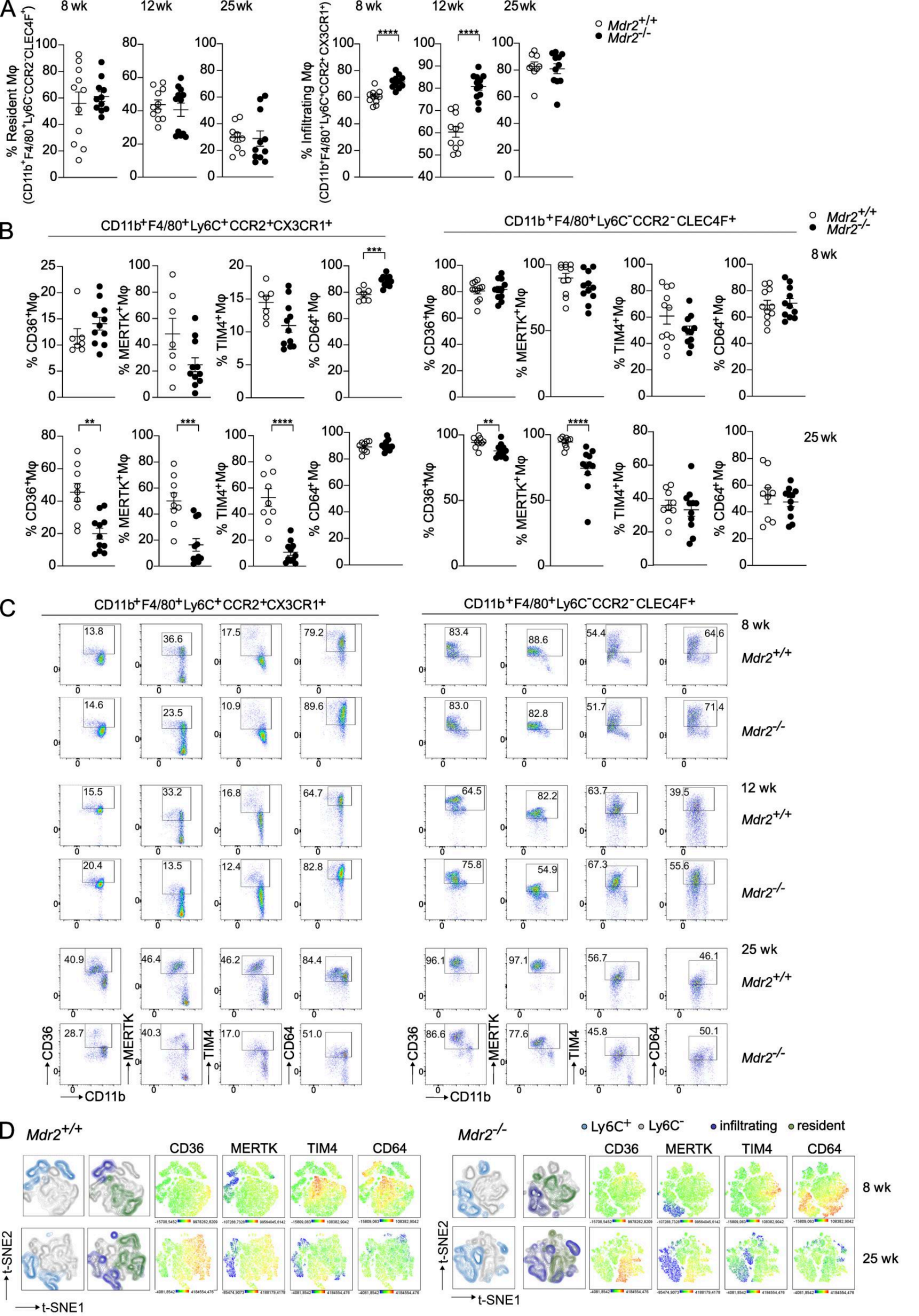
**Phagocitic profile of hepatic** **Mφ** **during cholangitis progression in *Mdr2*** ^**−/−**^ **mice** **.** **(A)** Graphs representing the frequencies of resident and infiltrating Mφ in *Mdr2*^*−/−*^ and *Mdr2*^*+/+*^ mice at 8, 12, and 25 wk of age. Two independent experiments were performed. Each data point represents one independent sample. *n* = 9–11; mean ± SEM. Mann–Whitney U test. **(B and C)** (B) Pooled data and (C) representative FACS plot reporting frequency of resident and infiltrating Mφ expressing the phagocytic receptors CD36, MERTK, TIM4, and CD64 isolated from the liver of *Mdr2*^*+/+*^ (clear dots) and *Mdr2*^*−/−*^ (filled dots) mice at 8 and 25 wk of age. *n* = 9–12 samples/time point. Mean ± SEM. Mann–Whitney U test. **(D)** t-SNE maps showing the expression of the phagocytic receptors CD36, MERTK, TIM4, and CD64 in infiltrating and resident Mφ within the Ly6C^+^ and Ly6C^−^ clusters in 8- and 25-wk-old *Mdr2*^*+/+*^ and *Mdr2*^*−/−*^ mice. **P < 0.01; ***P < 0.001; ****P < 0.0001.

To investigate the role of efferocytosis exerted by Mφ during the course of inflammation of the bile duct system, we first analyzed the expression of different phagocytic receptors in Mφ populations isolated from the liver of multidrug resistance protein 2 knock-out mice (*Mdr2*^*−/−*^) and controls (*Mdr2*^*+/+*^*)* during different phases of cholangitis progression. The *Mdr2*^*−/−*^ mouse is an established experimental model of chronic biliary liver disease mirroring some of the pathological features of patients with PSC. *Mdr2*^*−/−*^ mice carry a mutation in *Abcb4*, the gene encoding for canicular phospholipid transporter, which causes the absence of phosphatidylcholine from bile, thus leading to cholangitis development (Popov et al., 2005; Fickert et al., 2004). Here, liver-resident (CD45^+^Ly6G^−^CD11b^+^F4/80^+^Ly6C^−^CCR2^−^CLEC4F^+^) and -infiltrating (CD45^+^Ly6G^−^CD11b^+^F4/80^+^Ly6C^+^CX3CR1^+^CCR2^+^) Mφ populations (Guilliams et al., 2022; Yona et al., 2013; Nusse and Kubes, 2025) from female mice, which develop more severe hepatobiliary damage than male mice (Li et al., 2017), were analyzed (Fig. 1 A). No differences in the frequency of liver-resident Mφ during disease progression were detected in *Mdr2*^*−/−*^ mice compared with the control counterparts. Only an increase in the percentage of Mφ infiltrating the *Mdr2*^*−/−*^ livers at 8 and 12 weeks (wk) of age were observed compared with the corresponding population in the *Mdr2*^*+/+*^ livers (Fig. S1 A). Conversely, analysis of the expression of the phosphatidylserine (PtdSer)-dependent phagocytic receptors CD36, MERTK, and TIM4 and the IgG Fc receptor I CD64 at different disease stages (*Mdr2*^*−/−*^ mice at 8, 12, and 25 wk of age) reveals alterations of phagocytic receptor expression on both Mφ populations (Fig. 1 B and Fig. S1 B).

In particular, no major differences were observed at early stages (8 wk) of disease progression (Fig. S1, B–D). In contrast, at later stages (12 and 25 wk of age), the class B scavenger receptor CD36 and the tyrosine kinase receptor MERTK displayed differential regulation between Mφ populations. MERTK was consistently downregulated in both populations at both 12 and 25 wk of age, whereas CD36 was markedly downregulated exclusively in resident Mφ at both 12 and 25 wk of age. In contrast, TIM4 shows a trend toward reduced expression in the infiltrating population at several of the analyzed time points, while CD64 expression was substantially increased in both populations, particularly at 12 wk of age (Fig. 1 B; and Fig. S1, B and C). Interestingly, the receptor pairs MERTK and CD36, as well as TIM4 and CD64, appeared to be frequently co-expressed and differentially distributed across Mφ subclusters, as indicated by the t-distributed Stochastic Neighbor Embedding (t-SNE) plots (Fig. 1 C and Fig. S1 D).

**Figure S2.**
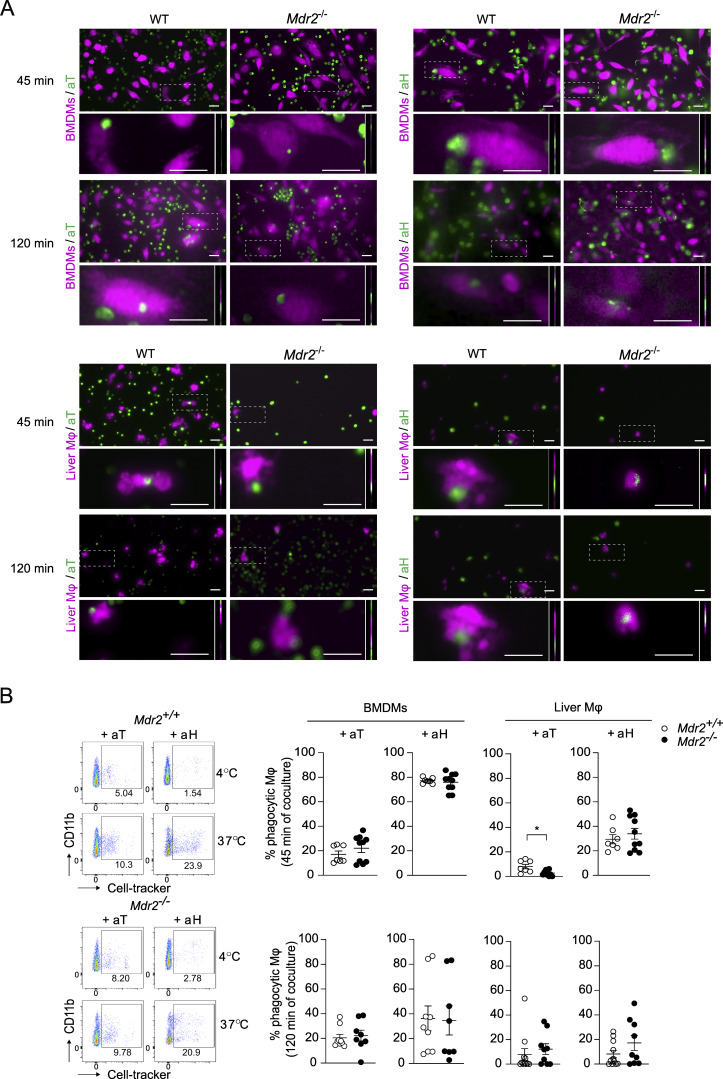
**Efferocytic** **capacity of bone marrow-derived and hepatic Mφ from *Mdr2*^*−/−*^ mice.** **(A and B)** (A) Representative images, and (B) FACS plots from liver Mφ and graphs reporting the frequency of *Mdr2*^*+/+*^ and *Mdr2*^*−/−*^ Mφ differentiated from the bone marrow (BMDMs) or isolated from the liver (liver Mφ), engulfing in vitro apoptotic thymocytes (aT) or apoptotic hepatocytes (aH) upon 45 min or 120 min coculture. Efferocytic Mφ are identified as CD11b^+^F4/80^+^ cells, expressing the apoptotic cell tracker. Scale bar, 20 μm. *n* = 7–10; mean ± SEM, Mann–Whitney U test. *P < 0.05.

In line with the alterations in PtdSer-dependent phagocytic receptor expression observed and the damage occurring in the livers of the *Mdr2*^−/−^ mice (Krishnan et al., 2020), accumulation of different dying cell types was detected, with around 60% of neutrophils (CD45^+^Ly6G^+^) and T cells (CD45^+^CD3^+^) and around 40% of parenchymal cells (CD45^−^) being Annexin-V^+^ in the damaged liver of *Mdr2*^*−/−*^ mice at 12 wk of age (Fig. 1 D). We next examined the capability of *Mdr2*^−/−^ Mφ isolated from liver and bone marrow, as representative of resident and infiltrating Mφ populations, to phagocytose apoptotic parenchymal and non-parenchymal cells (e.g., apoptotic hepatocytes [aH] and apoptotic thymocytes [aT] cells) in vitro. Efferocytic capacity was evaluated via flow cytometry as the difference between the percentage of Mφ (CD11b^+^F4/80^+^) binding (4°C) and taking up (37°C) the cell tracker–labeled apoptotic cells as well as by imaging (Fig. S2, A and B). Mφ isolated from the bone marrow and the liver of *Mdr2*^−/−^ mice showed a similar capacity to their WT counterparts to engulf either aT and aH, with only a transient reduction in *Mdr2*^−/−^ liver Mφ phagocyting aT but not aH after 45 min of coculture (Fig S2 B).

To evaluate the features of hepatic Mφ undergoing regulation of efferocytic receptor expression during cholangitis, we FACS-sorted liver CD45^+^Ly6G^−^CD11b^+^F4/80^+^ Mφ from 12-wk-old *Mdr2*^−/−^ mice based on the expression of the two PtdSer-dependent phagocytic receptors MERTK and CD36 (Fig. 1 E) to obtain two distinct populations defined as phagocytic receptor–enriched and –deficient Mφ. In line with the reduced frequency of hepatic Mφ expressing the efferocytic receptors in *Mdr2*^−/−^ mice, we FACS sorted fewer phagocytic receptor–expressing cells (average of 44,057 cells/liver) than their non-expressing counterparts (average of 226,878 cells/liver) (Fig. 1 E). We then performed lipidomic analysis via high-performance liquid chromatography coupled with electrospray ionization tandem mass spectrometry (HPLC–ESI–MS/MS) and identified the phagocytic receptor–enriched Mφ as the population retaining higher amounts of both conjugated and unconjugated bile acids (Fig. 1 F). Interestingly, this subpopulation of hepatic Mφ also show increased levels of *Lxr*a (Fig. 1 G), a nuclear receptor that functions to prevent bile acid toxicity (Uppal et al., 2007) as well as to regulate Mφ phagocytic capacity and its immune consequences (Alonso Gonzalez et al., 2009).

We then assessed the expression of different markers that define the identity and polarization status of phagocytic receptor–enriched and –deficient Mφ in the livers of *Mdr2*^−/−^ mice. We observed an increase in the expression of CCR2 in the phagocytic receptor–enriched Mφ compared with the –deficient counterpart (Fig. 1 H). CCR2 expression suggests that these Mφ may be enriched in cells recruited from the circulation rather than as a result of replication of liver resident Mφ, i.e., Kupffer cells (KCs) (Tsou et al., 2007; Serbina and Pamer, 2006). These Mφ also exhibited a higher antigen presentation capacity as indicated by elevated levels of MHC-II expression but also reduced CX3CR1 expression, which has been associated with an increased fibrotic potential in a model of carbon tetrachloride–induced liver inflammation (Aoyama et al., 2010).

Additionally, the expression of the mannose receptor CD206 was strongly increased in the phagocytic receptor–enriched vs. –deficient Mφ (Fig. 1 H), featuring a bile acid responding Mφ population present in patients with PSC (Chen et al., 2019). Furthermore, the increased expression of CD206 may suggest a role for these cells in infiltrating the biliary epithelium or directly associating with its luminal side, as previously described for CD206^+^ cells in the large bile ducts of both mouse and human injured livers (Guicciardi et al., 2018).

**Figure S3.**
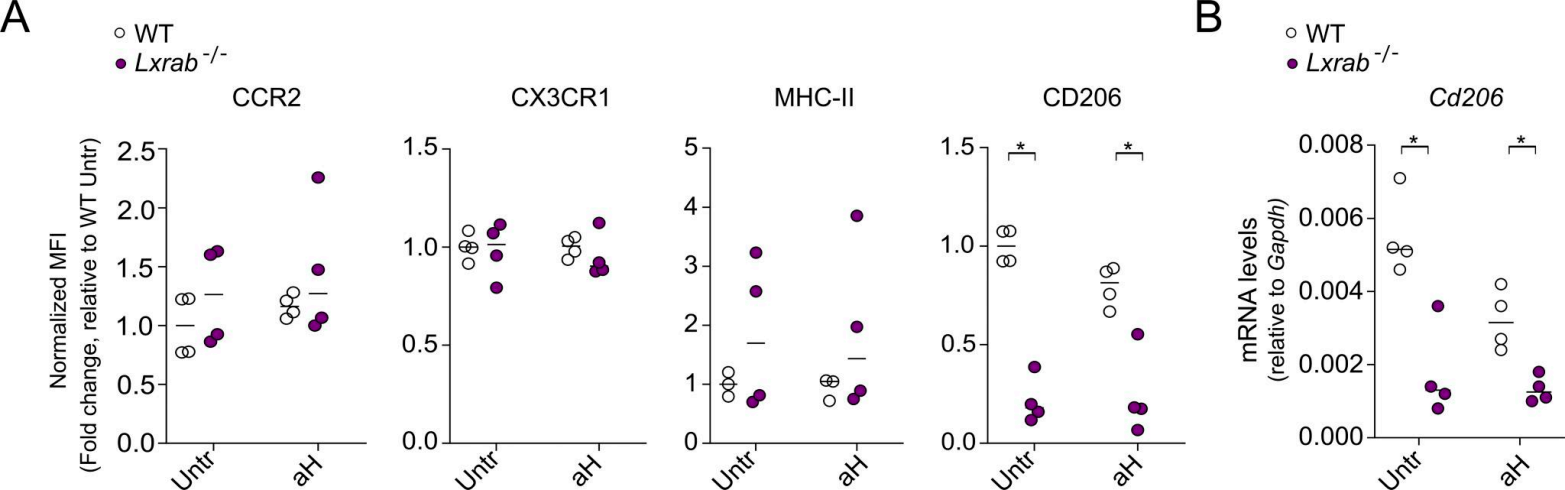
***Lxrab*-dependent regulation of Mφ phenotypical features.** BMDMs from either WT or *Lxrab*^−/−^ mice were left untreated (Untr) or treated for 45 min with apoptotic hepatocytes (aH).** (A)** After removal of the non-cleared aH, flow cytometry analysis on BMDMs was performed. Graphs reporting the normalized median fluorescence intensity (MFI) for CCR2, CX3CR1, MHC-II, and CD206 are shown. **(B)** mRNA level of *Cd206* in BMDMs treated as in A are shown. *n* = 4; mean ± SEM, Mann–Whitney U test. *P < 0.05.

Interestingly among the markers analyzed, only CD206 expression, unlike that of the other markers, showed a trend toward regulation by LXR*αβ* at both protein and mRNA levels, as observed in bone marrow–derived Mφ (BMDMs) isolated from WT or *Lxrab*^*−/−*^mice, either untreated (Untr) or exposed to aH (Fig. S3, A and B).

Collectively, these data indicate that, in the *Mdr2*^−/−^ livers, bile acids mainly accumulate within a population of CD45^+^Ly6G^−^CD11b^+^F4/80^+^MERTK^+^CD36^+^ efferocytic Mφ, which displays elevated *Lxra* expression. These Mφ are characterized by the enrichment in disease-associated Mφ markers during cholangitis.

### Bile acid retention is due to the uptake of bile acid–laden dying cells and influences Mφ efferocytic function

**Figure 2.**
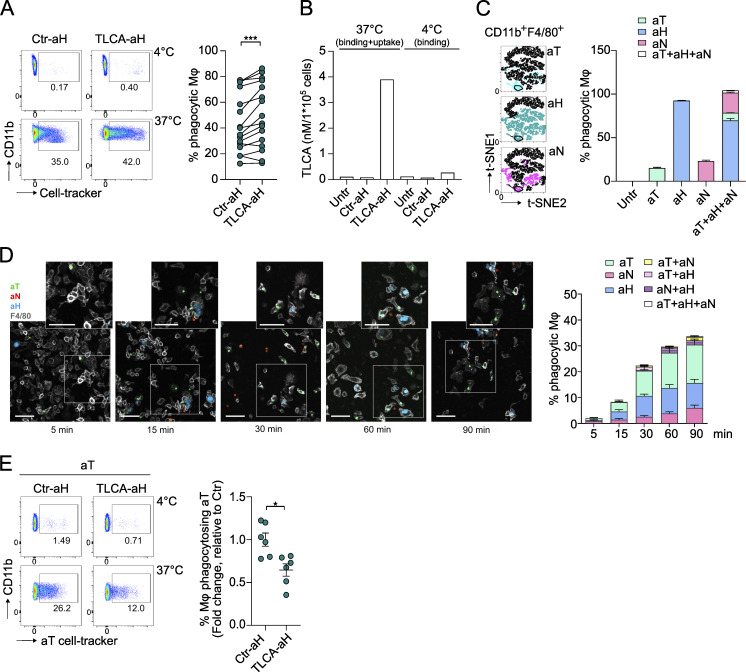
**Bile acid–laden apoptotic hepatocytes are a carriers of bile acids within the efferocytic Mφ. (A)** Apoptotic hepatocytes (aH) were treated with the vehicle DMSO as control (Ctr-aH) or with 50 µM of the secondary bile acid TLCA (TLCA-aH) for 75 min. aH were then added to BMDMs and cocultured with for 45 min. Representative flow cytograms and data reporting the frequency of Mφ phagocytosing Ctr-aH or TLCA-aH are shown. *n* = 16 biological replicates; mean ± SEM. Wilcoxon test. **(B)** Amount of TLCA detected in Mφ after uptake of TLCA-aH (37°C) or upon binding to TLCA-aH (4°C), as detected by HPLC. Bars represent pooled data from two independent experiments, each with three biological replicates per condition. **(C and D)** BMDMs were exposed to aH, apoptotic thymocytes (aT), or apoptotic neutrophils (aN) individually or all the three cell types concomitantly (aT+aH+aN). Apoptotic cells were stained with different cell dyes, and the phagocytic capacity of Mφ was assessed via flow cytometry or immunofluorescence. *n* = 3 biological replicates/condition. **(C)** t-SNE plots, concatenated from three mice/condition, report the fraction of Mφ that have been phagocytosing aT (in light blue), aH (in dark green), or aN (in pink), after 45 min of coculture. The black fraction in each t-SNE plot represents the non-phagocytic Mφ. The gate (black circle) shows the Mφ that have been phagocytosed aT, aH, and aN simultaneously. Representative t-SNE plots and quantification are reported. One representative experiment with BMDMs isolated from three WT mice is shown. **(D)** Immunofluorescence was performed on Mφ (F4/80, white) during the efferocytosis of aT (green), aH (blue), and aN (red). The frequency of phagocytic Mφ was analyzed 5, 15, 30, 60, and 90 min after adding the apoptotic cells to the Mφ culture. IF images representative of three independent experiments are reported. Quantification has been performed by counting a total of 16 different areas/condition. Scale bar, 50 μm. **(E)** Mφ treated as reported in A are further tested for their capacity to phagocyte apoptotic cells (aT) in vitro. Representative dot plots and pooled data reporting the frequency of Mφ phagocytosing aT in three independent experiments. *n* = 6 biological replicates; mean ± SEM. Mann–Whitney U test. *P < 0.05; ***P < 0.001.

We next wondered how bile acids can be retained within efferocytic Mφ and whether internalized bile acids may be affecting hepatic Mφ function. During cholangitis, parenchymal cells and primarily cholangiocytes have been proposed to undergo apoptosis due to bile acid accumulation (Perez and Briz, 2009). Whether these dying cells are engulfed and what their consequences are for efferocytic cells remain unknown. Our data show that hepatic Mφ from *Mdr2*^−/−^ mice exhibit an altered phagocytic receptor signature associated with intracellular accumulation of bile acids. Based on these observations, we hypothesize that efferocytosis of bile acid–laden apoptotic parenchymal cells may trigger bile acid accumulation within efferocytic Mφ and consequently affect their function. To test this hypothesis, we devised an in vitro experimental approach in which BMDMs were exposed to aH preloaded with 50 μM of the secondary bile acid TLCA (TLCA-aH) or not (i.e., treated with DMSO as a vehicle control, Ctr-aH). TLCA is known for its anti-inflammatory effects on Mφ (Wammers et al., 2018). In vitro, Mφ appear to properly engulf dying hepatocytes, even phagocytosing TLCA-treated aH more efficiently than their control aH (Ctr-aH) counterpart (Fig. 2 A). Lipidomic content of Mφ either exposed to aH or to TLCA-loaded aH was afterward analyzed via HPLC. Accumulation of lipids within Mφ occurs exclusively when they phagocyte aH carrying bile acids, and actual uptake (37°C) rather than binding to aH (4°C) is required for this accumulation to occur (Fig. 2 B).

During liver injury, hepatic Mφ encounter not only parenchymal cells but also immune cells undergoing cell death. Their clearance by efferocytic Mφ is essential for restoring liver homeostasis. In line with this, to evaluate the dynamic of the efferocytic process, we mimicked this situation in vitro by leaving BMDMs either untreated (Untr) or exposing them to aT alone; aH alone; apoptotic neutrophils (aN) alone; or a combination of aT, aH, and aN (aT+aH+aN). Mφ phagocytic capacity was then quantified via flow cytometry 45 min after the initiation of their coculture with the dying cells. Representative t-SNE plots and corresponding quantification suggest that at the time point analyzed, the majority of Mφ preferentially phagocytose one type of apoptotic cell at a time (Fig. 2 C). Only a small percentage (2.6 ± 1.5 %) of Mφ was able to phagocytose all different types of apoptotic cells simultaneously. These results were further confirmed via imaging. Representative immunofluorescence pictures and corresponding quantification confirm that during a 90-min interaction between Mφ and apoptotic cells, Mφ divide their tasks, and during the first 15 min, preferentially take up only one type of apoptotic cell at a time. Only at later time points (30, 60, and 90 min), a small fraction of Mφ acquire the ability to engulf multiple types of apoptotic cells (Fig. 2 D). These data suggest that although Mφ phagocytose both parenchymal and non-parenchymal cells, the concomitant uptake of different cell types follows distinct dynamics. Our data indeed indicate that Mφ have limited ability to simultaneously engulf cells with diverse identities at early time points, with broader phagocytic activity emerging only at later time points. Based on these findings, we next investigated whether the initial uptake of bile acid–loaded aH and the subsequent retention of bile acids within efferocytic Mφ affect their ability to engulf additional dying cells. To address this, Mφ that had phagocytosed either aH treated with a vehicle control (Ctr-aH) or TLCA-loaded aH (TLCA-aH) were subsequently exposed in vitro to aT. Notably, only Mφ preexposed to bile acid–loaded hepatocytes showed a reduced capacity to engulf additional dying cells, as aT (Fig. 2 E). Together, these results indicate that, in vitro, the engulfment of bile acid–laden apoptotic cells impairs subsequent Mφ efferocytic capacity.

### Engulfment of bile acid–laden dying cells shapes Mφ function

Next, we investigated the impact of bile acid retention on various Mφ immune functions.

**Figure 3.**
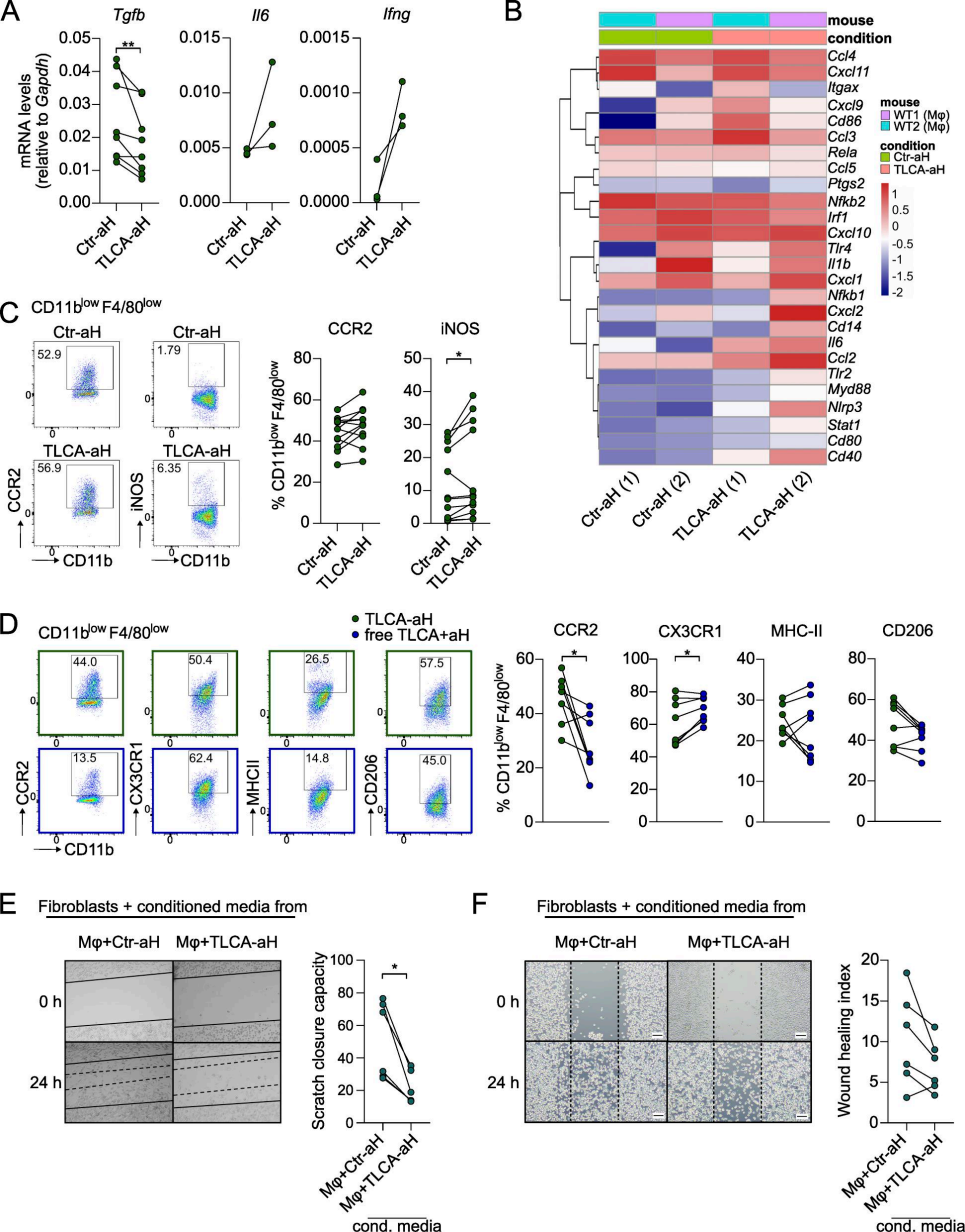
**Engulfment of bile acid–laden** **apoptotic hepatocytes** **shapes** **Mφ** **activation status.** **(A–C)** BMDMs were exposed for 45 min to apoptotic hepatocytes (aH), either treated with a vehicle control (Ctr-aH) or preloaded with TLCA (TLCA-aH). After 45 min, the medium was washed away, and the cells were kept in culture for an additional 24 h. On those BMDMs, (A) cytokine mRNA levels, detected by qPCR, *n* = 3–7 biological replicates, mean ± SEM, Wilcoxon test; (B) mRNA transcripts analyzed via bulk mRNA sequencing, *n* = 2 biological replicates; (C) CCR2 and iNOS, detected via flow cytometry (on a live, CD11b^low^F4/80^low^ Mφ population), were analyzed. *n* = 12 biological replicates, mean ± SEM, Wilcoxon test. **(D)** BMDMs were exposed for 45 min to aH, either preloaded with TLCA (TLCA-aH) or to untreated apoptotic hepatocytes in the presence of free TLCA (free TLCA+ aH). After 45 min, the medium was washed away, and cells were kept in culture for an additional 24 h. Representative FACS plot and corresponding bar graph reporting frequency of BMDMs (live, CD11b^low^F4/80^low^) expressing different polarization markers are shown. *n* = 8, mean ± SEM, Wilcoxon test. **(E and F)** Representative images and quantification of scratch closure capacity in fibroblasts (WEHI-164 cell line), either (E) creating a gap by mechanically damaging the cell layer or (F) using a culture insert, followed by treatment for 24 h with conditioned media isolated from WT BMDMs exposed for 45 min to aH, either treated with a vehicle control (Mφ+Ctr-aH) or preloaded with TLCA (Mφ+TLCA-aH). Dotted lines indicate the area covered by migration of the cells into the wound 24 h later. Scale bar, 100 μm. *n* = 6–8, mean ± SEM, Wilcoxon test. *P < 0.05; **P < 0.01.

We consistently observed reduced *Tgfb* mRNA expression in Mφ exposed to TLCA-loaded aH compared with those engulfing control aH. In contrast, when detectable, *Ifng* and *Il6* expression was increased in Mφ phagocytosing bile acid–loaded aH, suggesting an enhanced pro-inflammatory profile (Fig. 3 A).

Bulk RNA sequencing (RNAseq) of Mφ revealed increased expression of *Il6*, *Ccl2*, *Nlrp3*, and *Cd40* in Mφ engulfing TLCA-loaded aH compared with those exposed to aH treated with a vehicle control, although absolute expression levels remained low (Fig. 3 B). At the protein level, Mφ taking up TLCA-loaded aH showed a trend toward increased CCR2 and a statistically significant increase in iNOS expression, as determined by flow cytometry (Fig. 3 C).

**Figure S4.**
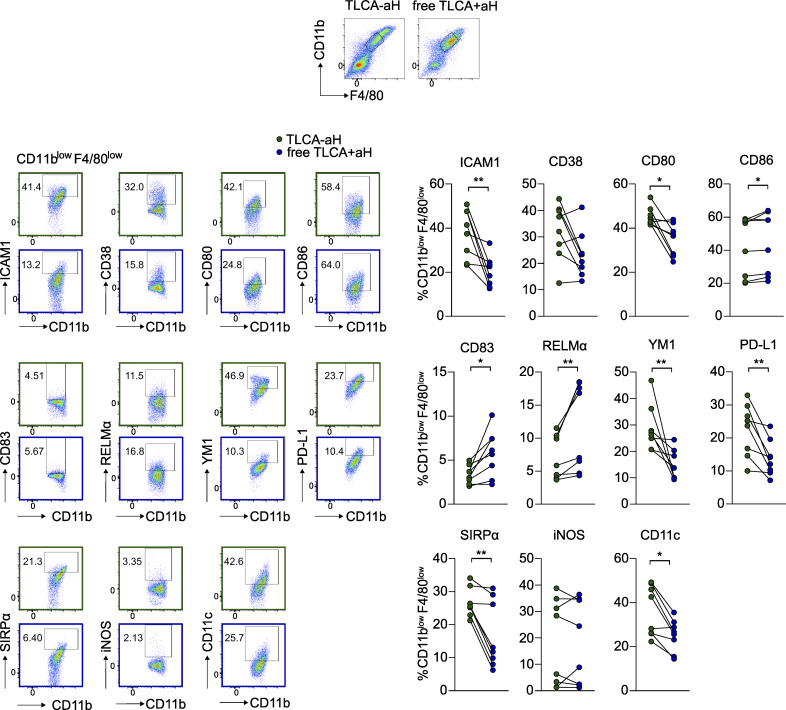
**Free TLCA and TLCA-loaded hepatocytes differentially impact Mφ phenotypic signature.** BMDMs were exposed for 45 min to either apoptotic hepatocytes (aH) pre-loaded with TLCA (TLCA-aH) or to untreated aH in the presence of free TLCA (free TLCA+aH). After 45 min, the medium was washed away, and cells were kept in culture for an additional 24 h. Gating strategy, representative FACS plot, and corresponding bar graph reporting frequency of BMDMs (live, CD11b^low^F4/80^low^) expressing different polarization markers are shown. *n* = 8, mean ± SEM, Wilcoxon test. *P < 0.05; **P < 0.01.

To determine whether bile acids act on Mφ only when carried by aH or also as free molecules, we exposed Mφ either to aH in the presence of free bile acids (free TLCA+aH) or to aH preloaded with bile acids (TLCA-aH). We then assessed Mφ phenotype by analyzing surface and intracellular polarization markers (Fig. 3 D and Fig. S4). Engulfment of bile acid–loaded hepatocytes caused marked changes in Mφ expression of both pro- and anti-inflammatory markers compared with Mφ exposed to free bile acids, therefore suggesting that the way in which bile acids are sensed contributes to their impact on target cells.

Additionally, given the role of fibroblasts in regulating parenchymal liver fibrosis, we evaluated the impact of Mφ efferocytosis of bile acid–laden aH on fibroblast function via two different scratch wound-healing assays. We added Mφ-conditioned media to a fibroblast monolayer after either creating a gap by mechanically damaging the cell layer or using a culture insert. Interestingly, results from both assays revealed that soluble factors released by Mφ, which have phagocytosed bile acid–laden aH, prevent fibroblast gap closure, compared with fibroblasts treated with conditioned media from Mφ taking up control aH (Fig. 3, E and F).

Although the specific soluble mediators responsible for the impaired fibroblast wound-healing capacity remain to be identified, these findings collectively suggest that bile acid uptake through efferocytosis induces a predominantly Mφ pro-inflammatory activation status, even in the absence of exogenous stimulation—an experimental strategy commonly used when investigating the effects of efferocytosis on Mφ profile (Voll et al., 1997; Bosurgi et al., 2017).

### aH laden with distinct bile acids lead to altered Mφ responses

Profiling of plasma bile acids in PSC patients reveals the accumulation of distinct bile acids, alterations in the conjugated fraction, and changes in primary-to-secondary bile acid ratios (Mousa et al., 2021). Given that bile acids differ in toxicity depending on their hydrophobicity, we next tested whether in vitro aH loaded with distinct bile acids, such as the conjugated ones taurocholic acid (TCA), taurochenodeoxycholic acid (TCDCA) and glycochenodeoxycholic acid (GCDCA), could differentially affect Mφ responses upon their engulfment.

**Figure 4.**
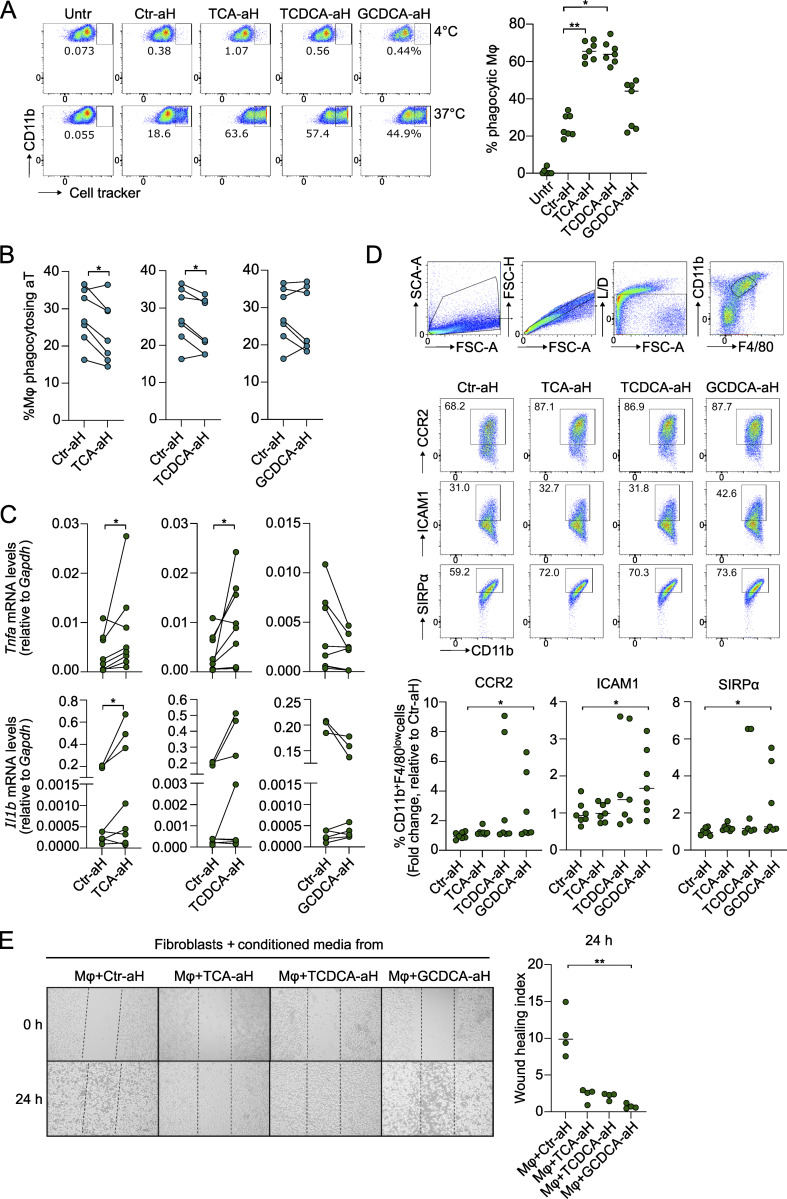
**Efferocytosis of hepatocytes loaded with distinct bile acids alters** **Mφ** **responses *in vitro*.** BMDMs were left untreated (Untr) or exposed to aH that were either treated with a vehicle control (Ctr-aH) or preloaded with different bile acids (TCA, TCDCA, and GCDCA). **(A–E)** Efferocytosis efficiency is shown through representative FACS plots and a bar graph reporting pooled data of BMDMs phagocytosing aH for 45 min at either 4°C (binding only) or 37°C (binding + uptake). *n* = 7 biological replicates, mean ± SEM; Friedman test followed by Dunn’s multiple comparison. BMDMs exposed to aH treated as described in A were then rested for 2 h and either (B) subsequently exposed to additional dying cells (aT) for 45 min and their efferocytosis efficiency analyzed; *n* = 7 biological replicates, mean ± SEM, Wilcoxon test; or (C) *Tnfa* and *Il1b* mRNA expression quantified by qPCR; *n* = 7, mean ± SEM, Wilcoxon test; or (D) analyzed for phenotypic marker expression after 24 h by flow cytometry (on a population of live, CD11b^+^F4/80^low^ Mφ); *n* = 7 biological replicates, mean ± SEM, Friedman test followed by Dunn’s multiple comparison test; or (E) analyzed for their capacity to induce wound closure in fibroblasts. A culture insert was added to a fibroblast layer, and then fibroblasts were exposed to Mφ-conditioned media (collected from Mφ treated as in A) for 24 h. *n* = 4 biological replicates, mean, ±SEM, Friedman test followed by Dunn’s multiple comparison test. *P < 0.05; **P < 0.01.

Consistent with our previous observations using TLCA-loaded aH (Fig. 2 A), BMDMs exhibited increased efferocytic capacity when exposed to aH loaded with TCA, TCDCA, but not GCDCA (Fig. 4 A). Moreover, uptake of aH loaded with TCDCA or TCA impaired the Mφ capacity to engulf additional dying cells, as previously observed with TLCA (Fig. 4 B).

Uptake of aH loaded with TCA was associated with increased expression of both *Tnf*a and *Il1b* mRNA in Mφ (Fig. 4 C). Similarly, TCDCA increased *Tnfα* expression, whereas GCDCA reduced it by trend. At the phenotypic level, among the markers analyzed, we only detected differences in Mφ that phagocytosed hepatocytes loaded with GCDCA, which showed increased expression of CCR2, ICAM1, and SIRPα (Fig. 4 D).

Finally, consistent with our previous findings with TLCA, all bile acids analyzed, when loaded into aH, resulted in a marked reduction in fibroblast wound-healing capacity in vitro (Fig. 4 E).

All in all, these data indicate that the uptake of hepatocytes loaded with distinct bile acids alters Mφ responses in vitro, albeit to different extents.

Notably, this effect occurs independently of bile acid water solubility and toxicity, suggesting that bile acids contained within aH induce Mφ phenotypic and functional reprogramming following engulfment.

### In PSC patients, hepatic Mφ enriched in hepatocyte transcripts exhibit a reduced phagocytic signature

**Figure 5.**
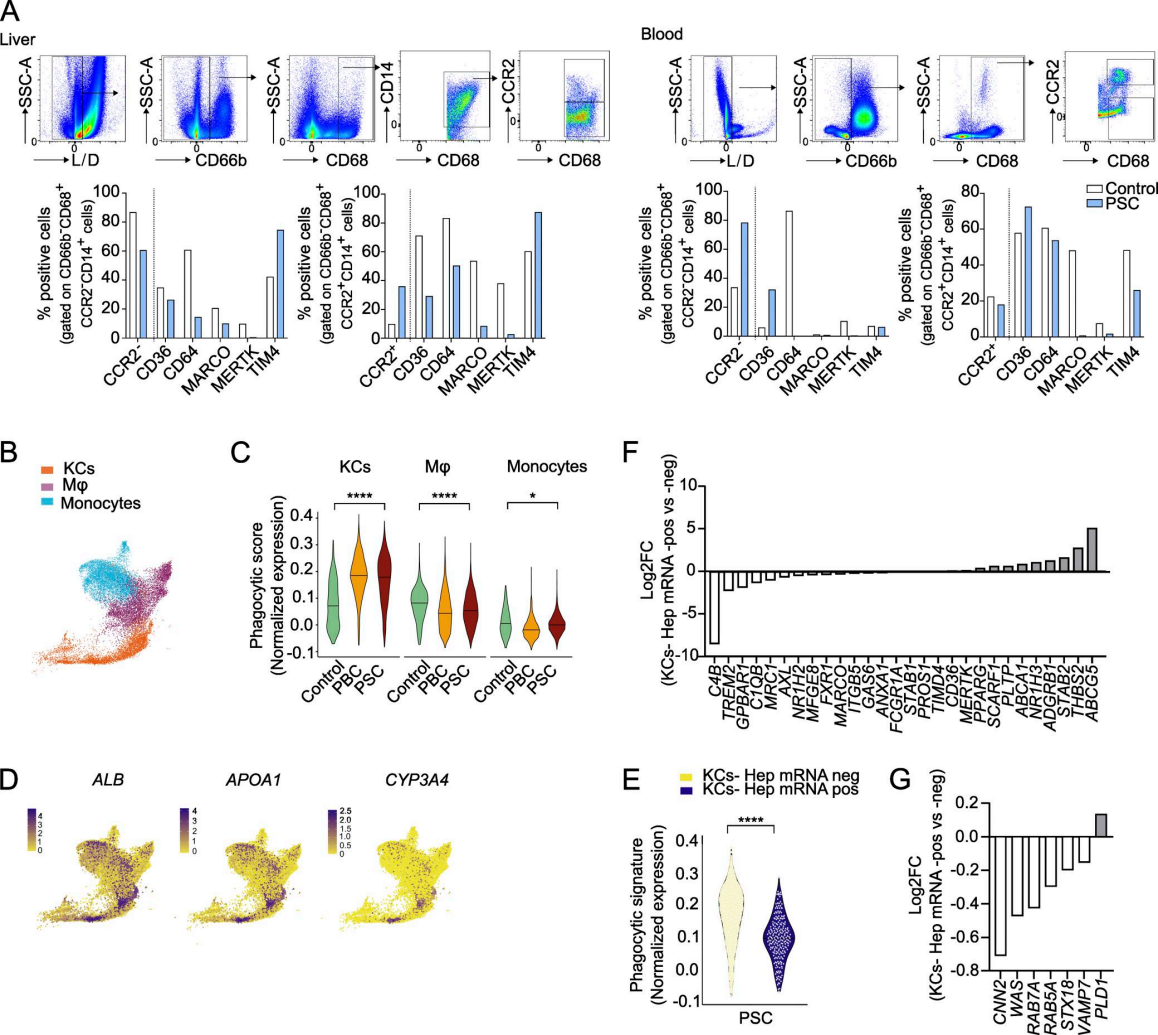
**Kupffer cells from PSC patients show enrichment in hepatocyte transcripts and reduced phagocytic signature.** **(A)** FACS plots reporting gating strategy for analysis of CD66b^−^CD68^+^CD14^+^CCR2^+^ and CD66b^−^CD68^+^CD14^+^CCR2^−^ Mφ/monocytes isolated from the liver and the blood of a PSC patient and a non-PSC patient control (Control). Bar graph reporting the frequency of cells expressing different efferocytic/scavenger receptors is shown. *n* = 1 patient/condition. **(B)** UMAP of Kupffer cells (KCs), Mφ, and monocytes from scRNAseq data from Andrews et al. (2024) of six normal (control), two PBC, and eight PSC livers. **(C)** Normalized expression score of phagocytic gene signature for different cell types across conditions. The horizontal bar of violin plot indicates the median. P value indicates Wilcoxon signed-rank test. **(D)** Normalized gene expression of selected genes on UMAP from B. **(E)** Normalized expression score of phagocytic gene signature for KCs from PSC patients either expressing (KCs-Hep mRNA pos) or not (KCs-Hep mRNA neg) hepatocyte-associated mRNA (*ALB*,* APOA1*, and *CYP3A4*). The horizontal bar of violin plot indicates the median. P value indicates Wilcoxon signed-rank test. **(F and G)** Waterfall plots displaying the log2 fold change (Log2FC) in expression of (F) phagocytosis-associated and LXR-dependent genes and (G) phagosome maturation-associated genes in KCs-Hep mRNA pos compared with KCs-Hep mRNA neg from PSC patients. *P < 0.05; ****P < 0.0001.

**Figure S5.**
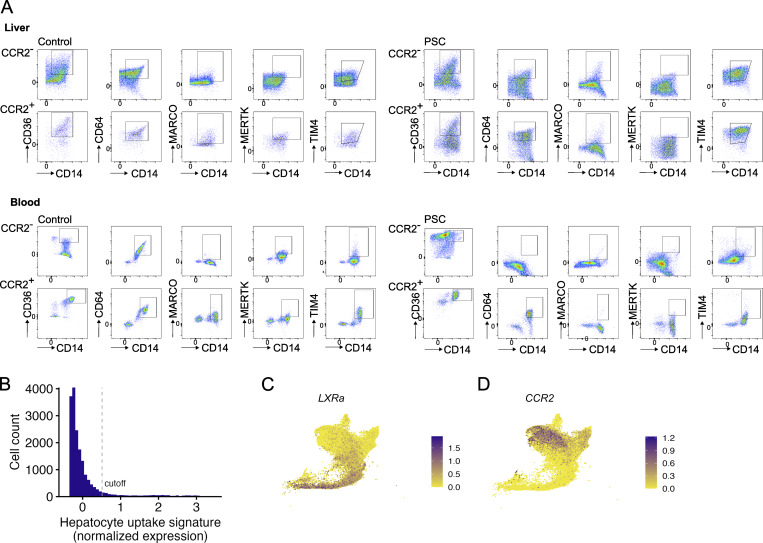
**Blood-derived and hepatic** **Mφ characterization in a PSC patient and a non-PSC control.** **(A)** FACS plots reporting gating strategy for analysis of CD36, CD64, MARCO, MERTK, and TIM4 in CD66b^−^CD68^+^CD14^+^CCR2^−^ and CD66b^−^CD68^+^CD14^+^CCR2^+^ Mφ/monocytes isolated from the liver and the blood of a non-PSC patient (Control) and a PSC patient. Analysis of Kupffer cells (KCs), Mφ, and monocytes from scRNAseq data from Andrews et al. (2024) of healthy, PBC, and PSC livers. **(B)** Histogram of normalized expression of hepatocyte uptake signature comprising *ALB*, *CYP3A4*, and *APOA1*. Cutoff indicates the 90^th^ percentile. **(C and D)** Normalized gene expression of (C) *LXRa* and (D) *CCR2* on UMAP from Fig. 5 B.

We next wondered whether an alteration in phagocytic capacity due to bile acid sensing could be observed in hepatic Mφ from PSC patients. To this end, Mφ were isolated from liver explants obtained from a patient with PSC and from the healthy portion of liver tissue obtained from a non-PSC patient with a liver adenoma, defined as a control (Control). Blood samples from the same patients were also collected. Within the CD14^+^CD68^+^ Mφ compartment, both the CCR2^+^ subset and the CCR2^−^ counterpart were present in comparable frequency in healthy and PSC liver biopsies (Fig. 5 A). In both Mφ subsets in the liver, although more prominently in the CCR2^+^ cluster, we detected reduced expression of the PtdSer receptors CD36 and MERTK, as well as the Fcγ receptor CD64, relative to their control counterpart. TIM4 expression, however, appeared to follow the opposite trend (Fig. 5 A and Fig. S5 A). Interestingly, a similar trend for MERTK and CD64 was observed in monocytes isolated from the peripheral blood of the same patients, with the exception of CD36, whose expression appeared to be higher in the blood of a PSC patient compared with the healthy control (Fig. 5 A and Fig. S5 A).

Given the exploratory nature of the human data due to the limited sample size, we further explored these findings by analyzing recently available single-cell/nucleus RNA sequencing data from 16 livers, including those from healthy individuals, PBC, and PSC patients (Andrews et al., 2024). Our analysis focused on KCs and hepatic Mφ/monocytes (Fig. 5 B). Across the three cell populations, we first analyzed the expression of a series of markers associated with Mφ phagocytosis, which defined our phagocytic score (Table S1). KCs show the highest phagocytic score compared with hepatic Mφ and monocytes in conditions such as PBC and PSC, where most likely, liver damage leads to higher dying cells accumulation compared with the healthy/control liver (Fig. 5 C). Next, we examined in the three clusters (KCs, hepatic Mφ, and monocytes) the expression of classical hepatocyte markers such as *ALB*, *CYP3A4*, and *APOA1* as potential indicator of the “passenger” mRNA transcripts originating from the engulfed hepatocytes, as previously described in different experimental settings (Lantz et al., 2020; Liebold et al., 2024). Interestingly, we detected all three hepatocyte genes primarily in KCs (Fig. 5 D and Fig. S5 B), likely as a result of the occurred efferocytosis. Accordingly, *LXR*a mRNA was mainly detected in KCs (Fig. S5 C).

Among the three distinct populations, KCs were characterized by absence of CCR2 in contrast to hepatic Mφ and monocytes (Fig. S5 D).

Hence, we focused on KCs from PSC livers and further categorized them based on the expression of hepatocyte mRNAs. Their phagocytic signatures were analyzed and compared between KCs expressing the hepatocyte mRNA (KCs-Hep mRNA pos) and those not expressing it (KCs-Hep mRNA neg). Interestingly, in PSC patients, the expression of hepatocyte mRNA transcripts, which may indicate uptake of dying hepatocytes, is associated with a reduced phagocytic score, mainly defined by decreased expression of genes related to “dying-cell-recognition” (e.g., *C4B*, *TREM2*, *C1QB*, and *AXL*) (Fig. 5 F) and associated with phagosome maturation in KC-Hep mRNA pos vs. KC-Hep mRNA neg, as shown in the waterfall plot (Fig. 5 G). An exception to this pattern is observed for *CD36* and *MERTK*, whose expression remains comparable between the two clusters, as well as the increased expression observed in LXR target genes (e.g., *PLTP*,* ABCG5*) in KC-Hep mRNA pos vs. KC-Hep mRNA neg (Fig. 5 F). This suggests that, despite previous uptake of hepatocyte material, KC-Hep mRNA pos cells in PSC patients may have a diminished capacity for further efferocytosis, thus supporting our in vitro data on murine Mφ impaired in their efferocytic ability upon uptake of bile acid–laden hepatocytes.

## Discussion

Previous studies have shown that bile acids act as regulatory molecules, dampening the inflammatory response in multiple cell types. Free bile acids in the environment signal through surface receptors such as TGR5 and via nuclear receptors like the farnesoid X receptor, pregnane X receptor, constitutive androstane receptor, vitamin D receptor, and small heterodimer partner. Their sensing leads to regulation of cytokines and chemokine secretion in both immune and non-immune cells (Fiorucci et al., 2021). Intestinal epithelial cells, for instance, respond to bile acids by increasing the expression of TGF-β and IP-10, decreasing the expression of pro-inflammatory mediators such as TNF-α, IL-8, and RANTES and reducing the production of PGE2 (Mencarelli et al., 2011). Mφ responding to bile acids in vitro show a reduced pro-inflammatory response to LPS (Wammers et al., 2018), thus highlighting the role of bile acids as immunosuppressors. Similarly, bile acids prevent the early immune response in blood-derived Mφ and KCs by downregulation of IL-6, TNF, IL-12, and IL-1α (Keitel et al., 2008; Haselow et al., 2013).

However, while bile acids can be present freely in the environment, the induction of cholestatic liver injury starts with the accumulation of bile acids in parenchymal cells (Cai and Boyer, 2021). Given this, we tested the impact of bile acids, not sensed freely, but through the uptake of bile acid–laden dying cells, on efferocytic Mφ and the surrounding environment.

In our dataset, both hepatic-resident and -infiltrating Mφ during cholangitis show altered expression of phagocytic receptors over the course of the disease. Some of these alterations are associated with inflammaging, defined as low-grade inflammation occurring under physiological aging conditions (Aprahamian et al., 2008; Poon and Ravichandran, 2024) and indeed observed in our control mice over time. However, pronounced differences are evident in CLEC4F^+^ liver-resident Mφ during cholangitis compared with their control counterparts, indicating the emergence of a dysregulated pool of efferocytic Mφ in *Mdr2*^−/−^ mice. However, the underlying causes and the functional consequences of this altered efferocytic Mφ pool remained unexplored.

Lipid-associated Mφ are phagocytic cells whose lipid handling capacity has been described in recent years to contribute to various metabolic diseases (Xu et al., 2024; Jaitin et al., 2019). Phagocytosis of lipid-laden aH has been described to occur via TREM2 engagement in the context of metabolic dysfunction-associated fatty liver diseases (Wang et al., 2023; Liebold et al., 2023). During cholangitis, cholangiocytes are exposed to high levels of bile acids at their apical membrane (Xia et al., 2006), and this accumulation has been suggested to promote their cell death (Zhou and Anakk, 2022; Guicciardi and Gores, 2002). In particular, the accumulation of toxic bile acids within parenchymal cells has been described as a trigger of increased hepatocytes and cholangiocytes sensitivity to DR5 and TRAIL-mediated signals, leading to their apoptosis and significantly contributing to cholestatic liver injury (Higuchi et al., 2002; Takeda et al., 2008). While bile acids have been proposed to cycle between cholangiocytes and hepatocytes (Xia et al., 2006), their transfer from parenchymal cells to immune cells has not been analyzed. Here, our data indicate that in vitro–dying parenchymal cells, upon their engulfment by Mφ, act as Trojan horses, recycling bile acids with the efferocytic Mφ populations and consequently altering their functions.

Although in our hands, bile acid–accumulating efferocytic Mφ represent only a small fraction of the hepatic Mφ pool during cholangitis, this cell subset may nonetheless be biologically relevant due to its distinct functional and phenotypic properties.

Indeed, low-frequency Mφ subsets have been shown to perform specialized and nonredundant roles in tissue homeostasis and in various disease settings (Chakarov et al., 2019; Bleriot et al., 2021). In line, we found that the bile acid–accumulating efferocytic Mφ display distinct phenotypical features compared with their non-efferocytic Mφ counterparts, including increased expression of CCR2, a prototypical pro-inflammatory marker of Mφ recruited to the periductular niche by damaged cholangiocytes during cholangitis progression (Guicciardi et al., 2018). Similarly, in vitro uptake of bile acid–laden dying parenchymal cells by Mφ and the resulting intracellular accumulation of bile acids affected Mφ capacity to subsequently phagocytose dying immune cells. We postulate that this bile acid–induced impairment of efferocytosis has a detrimental effect on cholangitis progression. This is consistent with previously published data showing that Arid3a, a DNA-binding transcription factor, is upregulated in hepatic Mφ during cholestasis and aggravates cholestatic liver injury by preventing efferocytosis through negative regulation of MERTK expression (Chen et al., 2023).

Lastly, soluble factors released by Mφ retaining bile acids affected fibroblast migratory capacity to the wound site. Further studies are required to identify the class of mediators underlying this effect, which may regulate wound healing during cholangitis through Mφ-fibroblast cross talk. Moreover, defining the precise function and localization within the liver of this numerically limited population of bile acid–accumulating efferocytic Mφ in vivo during cholangitis will be essential to determine whether Mφ interactions with parenchymal cells represents a targetable process for therapeutic purposes.

Interaction between cholangiocytes and Mφ has already been suggested by the increased amount of Mφ expressing the chemokine CCL28 in the liver peribiliary area in the early stage of PSC disease compared with other liver pathologies (Govaere et al., 2019) as well as in primary mouse cholangiocyte-derived organoids. Here, extracellular vesicles containing S100A11, released by activated cholangiocytes, stimulate pro-inflammatory cytokine expression in BMDMs (Katsumi et al., 2019). These data indicate that Mφ are actively involved in the interaction with cholangiocytes by exchanging a multitude of cues (Cadamuro et al., 2020). Our data shed light on efferocytosis as an additional mechanism at the basis of the interaction between parenchymal cells and efferocytic Mφ. Interestingly, only when bile acids are sensed through efferocytosis of bile acid–laden parenchymal cells, but not when present freely in the environment, do they detrimentally shape Mφ function. This indicates that the mode by which bile acids are sensed by Mφ critically determines their impact on target cells. Accordingly, further studies are required to delineate whether the recognition of bile acid–laden parenchymal cells occurs via classical “eat me” signals and how their uptake affects phagocytic receptor downstream signaling events, as, for instance, the activation of DNAX-activation protein 12 in the case of TREM2 engagement (Kober and Brett, 2017), with TREM2 being described to be involved in uptake of dying hepatocytes (Wang et al., 2023; Liebold et al., 2023). In conclusion, our data suggest that the uptake of bile acid–enriched dying cells triggers a cascade of events associated with altered Mφ function, including impaired efferocytic capacity and the potential consequent accumulation of apoptotic immune cells. All together these events may contribute to a sustained inflammatory response. Of note, in line with our murine data, human KCs from PSC patients, which display enrichment of genes typically associated with hepatocytes, likely reflecting mRNA derived from engulfed cells, exhibit a reduced phagocytic transcriptional signature. However, we were unable to detect a concomitant increase in pro-inflammatory gene expression at transcriptomic level. Based on this, further studies are required to define the functional role of this Mφ population in the injured liver and to determine whether, at specific stages of disease, these cells accumulate bile acids as a consequence of efferocytosis.

Hence, a more comprehensive understanding of the mechanistic events underlying the initial detrimental interaction between bile acid–laden parenchymal cells and efferocytic Mφ will assist in the development of future strategies for preventing disease progression and chronicity in liver pathologies. Furthermore, it may help to elucidate novel cellular mechanisms contributing to rapid liver cirrhosis development, as, for instance, in the context of cholangiocarcinoma.

Overall, the present study provides a captivating starting point for rethinking the role of bile acids and efferocytosis as anti-inflammatory and immunosuppressive triggers in the context of autoimmune liver diseases.

## Materials and methods

### Cell culture

All cell culture experiments were performed under sterile conditions. All cells were cultured at 37°C, 5% CO_2_, except for the Hepa 1–6 cell line (ATCC no. CRL-1830, RRID:CVCL_0327, here referred to as hepatocytes) that were maintained at 37°C 5–9% CO_2_. The WEHI-164 cell line (murine skin fibroblast, RRID:CVCL_2251), cultured in RPMI1640 with 10% FCS, 1% 200 mM L-glutamine, and 0.5% gentamicin, was used for the in vitro scratch assay. Cell lines were initially tested and confirmed negative for mycoplasma contamination.

### Mice

C57BL/6 WT mice (RRID:MGI:2159769) were bred and kept in a specific pathogen–free facility at the Bernhard Nocht Institute for Tropical Medicine as well as the University Medical Center Hamburg Eppendorf. *Mdr2*^*−/−*^ mice (B6.129P2-Abcb4tm1Bor/J RRID:IMSR_JAX:002539) were previously obtained from Jackson Laboratory. All mice were bred and housed with 12-h light/dark cycles, either at the animal care facility of the University Medical Center Hamburg-Eppendorf with access to standard chow diet (1318 rodent diet, Altromin) and water available ad libitum or at the Bernhard Nocht Institute for Tropical Medicine. The *Mdr2*^*−/−*^ mouse model was described previously (Fickert et al., 2004). Animal care was performed in accordance with governmental and institutional guidelines. All experiments complied with the Animal Research: Reporting of In Vivo Experiments guidelines and were approved by the local review board of the State of Hamburg, Germany (Behörde für Justiz und Verbraucherschutz).

*Mdr2* ^−/−^ and *Mdr2*^+/+^ 8–25-wk-old female mice were used for the reported experiments (Li et al., 2017); Mice were anesthetized with (CO_2_/O_2_) and sacrificed by an overdose of CO_2_ followed by a cervical dislocation in the absence of signs of respiration or corneal and interdigital reflexes. No animals were excluded from the study. Mice of different genotypes were co-housed in the same cages but were grouped according to genotype for experimental analyses. Within each genotype group, animals were randomly assigned to experimental conditions.

### Differentiation of BMDMs

BMDMs were obtained from mouse femurs and tibias. The bones were removed, disinfected with 70% ethanol (EtOH) for 30 s, and cut open at one epiphysis. The bones were then placed in 0.5-ml perforated tubes, then in 1.5-ml tubes, and centrifuged at 13,000 rpm for 2 min. Bone marrow cell suspension was then resuspended in 0.5 ml RPMI and filtered through a 40-μm cell strainer. Cells were then seeded in 15-cm Petri dishes in 10 ml RPMI containing 20% FCS, 30% L929-cell line supernatant, 2.5% L-glutamine, and 0.5% gentamycin. To differentiate the Mφ from the bone marrow–derived hematopoietic pluripotent stem cells, the medium was changed on day 3 by centrifugation at 300 × *g* for 5 min, and cells were resuspended in 10 ml fresh new medium. Cells were split into two new 15-cm dishes containing 10 ml of fresh medium on day 5. Cells underwent differentiation in BMDMs on day 7 and were seeded in 24-well plates at a concentration of 0.33 × 10^6^ cells/well for performing the different experiments. BMDMs from control and *Lxrab*^*−/−*^ (*Lxr*a/b double knockout) mice were obtained from femurs and tibias of WT and *Lxrab*^*−/−*^ mice housed at the University of Munster and kindly provided by Noelia A-Gonzalez.

### Isolation of neutrophils

Neutrophils were isolated from the bone marrow of >11-wk WT mice. A negative selection was performed via MACS-selection kit for neutrophils (Miltenyi) according to the manufacturer’s protocol.

### Hepa 1–6 cells and bile acid treatment

Hepa 1–6 cells were washed with PBS and detached using a cell scraper. For experiments where bile acid treatment took place, Hepa 1-6 were supplemented with 50 μM of the bile acid of choice (Taurolithocholic acid, TLCA [Steraloids C1470]; Taurocholic acid, TCA; Taurochenodeoxycholic acid, TCDCA; Glycochenodeoxycholic acid, GCDCA [Universitätsklinikum Hamburg-Eppendorf Biochemistry Department]) or DMSO (control) for ∼75–120 min before apoptosis induction. During the induction of cell death, cells were kept in media containing bile acids or DMSO. Afterward, apoptotic hepatocytes (aH) were washed, counted, and labeled (see Efferocytosis assay). Mφ were then co-cultivated for 45 min at 37°C and 4°C with bile acid– or DMSO-treated aH. Afterward, free apoptotic cells were washed out five times with PBS, and Mφ were then stained and analyzed at the flow cytometer (LSR II or Cytek Aurora).

### Induction of apoptosis

Apoptosis was induced in neutrophils (aN) by aging the cells for 24 h in cRPMI media containing a reduced amount of FCS (2.5–5% FCS) at 37°C, 5% CO_2_ in a 15-cm dish.

The thymus was isolated from WT mice and mashed over a 40-µm cell strainer. Apoptosis was induced in thymocytes (aT) by aging them for 24 h at 37°C, 5% CO_2_ in cRPMI media containing a reduced amount of FCS (2.5–5% FCS) in a 15-cm dish.

In hepatocytes (Hepa 1–6, aH), the apoptosis was induced by heating the cell suspension for 45 min at 41°C in PBS or in the bile acid or DMSO supplemented media with 0.5% FCS while shaking.

### Apoptotic cell staining with Annexin V and PI

For all cell types, the rate of apoptosis was measured by Annexin V and PI staining using an apoptosis detection kit (BioLegend). Cells were directly stained in FACS tubes containing 100 μl Annexin V binding buffer. 2 μl of Annexin V was added to stain phosphatidylserine (PtdSer) on the apoptotic cell surface. Cells were incubated for 15 min at room temperature (RT) in the dark. Next, 200 μl of Annexin V binding buffer and 1 μl of propidium iodide (PI) staining solution were added to the cell suspension, and the samples were measured directly at the flow cytometer.

### Efferocytosis assay

Mφ were seeded in two separate 24-well plates at a concentration of 0.33 × 10^6^ cells/well. One plate was placed on ice for 15 min at 4°C before starting the experiment. Two conditions of 37°C and 4°C were run simultaneously. At 37°C, Mφ are able to both bind and uptake apoptotic cells, while at 4°C, only binding to apoptotic cells can occur. Apoptotic cells were pre-labeled with an apoptotic cell dye and cocultured with Mφ for 45 min, in a ratio of 1:5 (aN and aT) or 1:2 (aH). Free apoptotic cells were then removed by washing with PBS five times. Efferocytic capacity (depicted as phagocytic Mφ) was either analyzed immediately by calculating the ratio between the frequency of Mφ labeled with the apoptotic cell dye at 37°C and 4°C or a second round of phagocytosis was performed after a resting time of 2 h. Phenotypic analysis by mRNA or FACS was performed after an additional 24 h of culture in fresh media. For some of the samples, analysis of the efferocytic capacity was performed via microscopy (see Imaging).

### Flow cytometry

Cells derived from mouse tissues were incubated with anti-CD16/CD32 (BioLegend) for 15 min at 4°C. To remove unbound antibodies during surface staining, cells were washed three times with 200 μl PBS/2% FCS per well (96-well plate). During the surface staining, antibodies were diluted in 25 μl of PBS/2% FCS/well and incubated for 35 min at 4°C in the dark. When intracellular epitopes were analyzed, a fixation with 100 μl of 1% paraformaldehyde (PFA) for at least 20 min at 4°C in the dark, followed by a permeabilization with 100 μl permeabilization buffer (BD Bioscience) for 15 min at RT, was performed. The intracellular antibodies were diluted in permeabilization buffer and then incubated for 45 min at 4°C in the dark. When liver samples were analyzed, cells were fixed and permeabilized using the Foxp3 staining Kit (eBioscience). The samples were analyzed at the LSRII or at the Cytek Aurora flow cytometer. In Fig. S3, one WT sample yielding a negative fluorescence intensity value (MHC-II) during Cytek Aurora analysis was excluded from the calculation. Data were analyzed with the FlowJo software (Tree Star RRID:SCR_008520), and FlowJo Version 10.01 was used to perform t-SNE analysis. Antibodies were purchased from commercial sources (Table 1).

**Table 1.** Mouse antibodies specifications

Epitope	Fluorochrome	Clone	Company	Cat.	RRID
Anti-rabbit IgG	BV421	Poly4064	BioLegend	406410	AB_10897810
Anti-goat IgG	FITC	Poly	TFS	A15964	AB_2534638
CCR2	BV650	SA203G11	BioLegend	150613	AB_2721553
CCR2	BV785	SA203G11	BioLegend	150621	AB_2721565
CCR7	BV421	4b12	BioLegend	120119	AB_10897811
CD115	BUV737	AFS98	BD Biosciences	750948	AB_2875029
CD11b	APC/Cy7	M1/70	BioLegend	101225	AB_830641
CD11b	AF700	M1/70	BioLegend	101222	AB_493705
CD11c	PE-Cy5	N418	BioLegend	117316	AB_493566
CD163	APC/Fire 810	S150491	BioLegend	155321	AB_2904294
CD206	PE-Dazzle	C068C2	BioLegend	141731	AB_2565931
CD36	AF488	HM36	eBioscience	53-0362-82	AB_2811851
CD38	PE-Fire700	90	BioLegend	102747	AB_2892264
CD40	APC	3/23	BioLegend	124611	AB_1134081
CD45	PE-Cy7	30-F11	BioLegend	103113	AB_312978
CD64	BV711	X54-5/7.1	BioLegend	139311	AB_2563846
CD64	BV605	x54-5/7.1	BioLegend	139323	AB_2629778
CD68	BV421	FA-11	BioLegend	137017	AB_2562949
CD80	SparkNIR 685	16-10A1	BioLegend	104761	AB_2924443
CD83	BV711	Michel-19	BioLegend	121529	AB_3662363
CD86	Pac. Blue	GL-1	BioLegend	105022	AB_493466
CLEC4F	APC	3E3F9	BioLegend	156803	AB_2814081
CX3CR1	BV785	SA011F11	BioLegend	149029	AB_2565938
CX3CR1	BV650	SA011F11	BioLegend	149033	AB_2565999
F4/80	AF700	BM8	BioLegend	123129	AB_2277848
F4/80	APC/Cy7	BM8	BioLegend	123118	AB_893477
ICAM1	BB700	3,00E+02	BD Biosciences	742100	AB_2871376
iNOS	BUV737	cxnft	eBioscience	367-5920-82	AB_2896022
Ly6C	BV570	HK1.4	BioLegend	128029	AB_10896061
Ly6G	BUV395	1A8	BD Biosciences	563978	AB_2716852
MERTK	PE	2B10C42	BioLegend	151505	AB_2617036
MERTK	RB780	108928	BD Biosciences	755415	​
MHCII	BV510	M5/114.15.2	BioLegend	107635	AB_2561397
MHCII	SparkBlue 550	M5/114.15.2	BioLegend	107661	AB_2876418
PD-L1	PE-Cy7	10F.9G2	BioLegend	124314	AB_10643573
RELMα	Unconjugated	Poly	PeproTech	500-P214	AB_1268707
SIRPα	PerCP-Cy5.5	P8A	BioLegend	144009	AB_2563547
TIM4	PerCP-eFluor710	54(RMT4-54)	eBioscience	46-5866-82	AB_2573781
YM1	Unconjugated	Poly	R&D	AF2446	AB_2079008

### Imaging

To analyze Mφ efferocytic capacity, Mφ were isolated from the respective tissue (bone marrow and liver) as described. After isolation, Mφ were stained with 15 µM Carboxyfluorescein diacetate succinimidyl ester (CFDA-SE) for 15 min at 37°C. The staining solution was then replaced with fresh media, and the cells were incubated for an additional 30 min at 37°C. Apoptotic cells were stained with 1 µM DeepRed for 30 min at 37°C before the staining solution was replaced. Mφ were then visualized using an inverted microscope (Leica Dmi8) equipped with 20×/0.40 N.A. PH1 objective. Images were recorded with an ORCA-Flash4.0 Digital camera (Hamamatsu Photonics) using the MetaMorph version 7.10.3.279 software (Molecular Device RRID:SCR_002368) or with a Spinning disk microscope (Nikon Eclipse TiE) equipped with a 40×/0.75 N.A. Plan Fluor Phase objective. Images were recorded with 2× Photometrics Prime 95B (back-illuminated sCMOS, 11-µm pixel size, 1,200 × 1,200 pixels) using VisiView version 4. All images were reconstituted by using Fiji (ImageJ RRID:SCR_002285) (Schindelin et al., 2012).

### Competitive efferocytosis assay

BMDMs were isolated and differentiated as described above (see Differentiation of BMDMs). The apoptotic cells, generated as described above (see Induction of apoptosis), were stained as follows: aT with 5 μM CFDA SE Cell Tracer (Invitrogen) in PBS for 15 min at 37°C, aN with 5 μM Cell tracker Red CMTPX (Invitrogen) in serum-free medium for 30 min at 37°C, and aH with 5 μM CellTrace Violet Dye (Invitrogen) in PBS for 15 min at 37°C. All apoptotic cell types were simultaneously co-cultured with the differentiated BMDMs in a 1:5 (aT, aN; 2.5 × 10^6^ cells/well) or 1:2 (aH, 1 × 10^6^ cells/wells) ratio for 5, 15, 30, 60, and 90 min to study the rate and the efficiency of the efferocytic process in the presence of different apoptotic cells. Afterward, cells were washed three times with 1× PBS to remove the remaining non-phagocytosed apoptotic cells and then fixed with 2% PFA in PBS for 5 min. Cover slips were then transferred to a wet chamber for staining of BMDMs, first with 2.5 μg/ml of rat anti-mouse F4/80 primary antibody (eBioscience RRID:AB_467558) at 4°C overnight and followed by incubation with 4 μg/ml of goat anti-rat Alexa Fluor 647 secondary antibody (Invitrogen RRID:AB_141778) for 1 h at RT, both in blocking solution (PBS/1% FCS). Cover slips were mounted on microscopy slides for imaging using Dako Fluorescence Mounting Medium.

### Competitive efferocytosis assay: Imaging and quantification

For quantification of efferocytic Mφ at different stages of phagocytosis, a Zeiss LSM800 confocal microscope was used to take images at 20× (quantification) and 40× magnification. 6–8 fields per time point were manually counted for Mφ phagocytosing single aT, aN, aH, or the combination of aT+aN, aT+aH, aN+aH, or aT+aN+aH. Results are shown as the percentage of efferocytic Mφ for each condition from the total of Mφ per field and time point.

### Scratch assay

To analyze the wound-healing capacity of fibroblasts, WEHI-164 cells were harvested, counted, and seeded at a density of 0.2 × 10^6^ cells/well in a 24-well plate with 1,000 μl of fibroblast medium. Once the cells had settled, the medium was removed, and one scratch was made across the fibroblast monolayer in the upper and lower halves of each well using a 200-μl pipette tip (Sarstedt). The wells were then gently washed with PBS to remove any detached fibroblasts. Mφ-conditioned medium was added to the fibroblasts, and each well was photographed using the EVOS FL Auto Imaging system (Thermo Fisher Scientific). All photographed positions were saved, and after a 24-h incubation at 37°C with 5% CO_2_, images were taken again. To determine the effect of supernatants from differently treated-Mφ, gap closure was calculated using ImageJ software (RRID:SCR_003070). In brief, three positions per scratch were selected, and the diameter of the scratch separating the fibroblasts was measured in the images taken at 0 and 24 h of cultivation. The average diameter for each time point was calculated, and gap closure was determined using the following formula: $gap\;closure\;\left[\%\right]=1-\frac{average\;\left(24\;h\right)}{average\;\left(0\;h\right)}\;x\;100$*.* Additionally, to investigate the wound-healing index, a second assay using a standardized barrier (Ibidi) to generate a defined gap was used. Briefly, 0.35 × 10^5^ fibroblasts were seeded in 70 μl of medium per Ibidi well and left to attach overnight. The inserts were then removed, the wells were gently washed, and the Mφ-conditioned media were added as described above. Three Images were acquired for each sample at 0 and 24 h using the LABSCOPE Imaging System (ZEISS) at 10× magnification. For analysis, the cell density within the gap area as well as in the regions immediately adjacent to the gap (left and right) was assessed, and the ratio between these densities was calculated. The mean of each sample was used, and the resulting values were normalized to the corresponding density ratio measured at 24 h.

### RNA isolation and quantitative real-time PCR analysis

To obtain RNA from cells after cell sorting (BD Aria, BD Biosciences), the Qiagen RNeasy mini Kit was used following the manufacturer’s instructions. The purity and amount of RNA were measured via NanoDrop2000c (Thermo Fisher Scientific). cDNA synthesis was performed with the iScript cDNA synthesis Kit (Bio-Rad). To quantify the specific DNA sequence in real-time, a quantitative real-time PCR (qPCR) was performed using the MaximaTM SYBR Green qPCR Master Mix (2X), with separate ROX vial (Thermo Fisher Scientific). The cDNA sequence was quantified and measured by the Rotor-gene 6000 machine or QuantStudio 3 (TFS). The reactions were performed in duplicates, with 40 ng cDNA and a final volume of 12 μl per reaction. Primer sequences are listed in Table 2. The analysis was performed with the Rotor-gene 6000 Series Software 1.7 or the DA2 software (TFS). *Gapdh* was used as a reference gene.

**Table 2.** Primer sequences for qPCR: Mouse primers

Name	Sequence 5′-> 3′
*Gapdh fw*	5′-TCC​CAC​TCT​TCC​ACC​TTC​GA-3′
*Gapdh rev*	5′-AGT​TGG​GAT​AGG​GCC​TCT​CTT-3′
*Cd206 fw*	5′-CTA​ACT​GGG​GTG​CTG​ACG​AG-3′
*Cd206 rev*	5′-GGC​AGT​TGA​GGA​GGT​TCA​GT-3′
*Ifnγ fw*	5′-TCA​AGT​GGC​ATA​GAT​GTG​GA-3′
*Ifnγ rev*	5′-TGA​GGT​AGA​AAG​AGA​TAA​TCT​GG-3′
*Il1b fw*	5′-GAA​GAA​GTG​CCC​ATC​CTC​TG-3′
*Il1b rev*	5′-AGC​TCA​TAT​GGG​TCC​GAC​AG-3′
*Il6 fw*	5′-TCC​AGT​TGC​CTT​CTT​GGG​AC-3′
*Il6 rev*	5′-GTG​TAA​TTA​AGC​CTC​CGA​CTT​G-3′
*Lxra fw*	5′-CAA​CAG​TGT​AAC​AGG​CGC​T-3′
*Lxra rev*	5′-TGC​AAT​GGG​CCA​AGG​C-3′
*Tfgb rev*	5′-TGA​CGT​CAC​TGG​AGT​TGT​ACG​G-3′
*Tgfb fw*	5′-GGT​TCA​TGT​CAT​GGA​TGG​TGC-3′
*Tnfα fw*	5′-AGC​CCC​CAG​TCT​GTA​TCC​TT-3′
*Tnfα rev*	5′-GAG​TTG​GAC​CCT​GAG​CCA​TA-3′

All oligonucleotides were synthesized and purchased at Eurofins.

### Genotyping of *Mdr2*^−/−^ and *Mdr2*^+/+^ mice

The genomic DNA was amplified by PCR using the DreamTaq PCR Kit (Thermo Fisher Scientific). An agarose electrophoresis was performed to evaluate the specific amplification using 1.5% agarose in TBE buffer. The PCR primers are depicted below.

Primer sequence:

*Mdr2* fwd: 5′-CCA​CAG​CCA​CAC​ACT​CAC​CT-3′

*Mdr2* Mut rev: 5′-CCA​GAC​TGC​CTT​GGG​AAA​AG-3′

*Mdr2* rev: 5′-CAT​CAA​ACC​ACG​TGC​AGA​AAA-3′

### Mouse tissue harvesting

Organs were harvested and collected in tubes containing PBS/2% FCS on ice prior to the processing for analysis.

### Isolation of Mφ from the liver

The mice were anesthetized with (CO_2_/O_2_) and sacrificed by an overdose of CO_2_ followed by a cervical dislocation in the absence of signs of respiration or corneal and interdigital reflexes. Perfusion was performed by inserting a butterfly needle into the vena cava and injecting 10 ml PBS to slowly remove blood. Livers were then harvested and stored in PBS/2% FCS on ice. After removing the gallbladder, the liver was minced and digested with 10 ml digestion buffer (DMEM, 1 mg/ml Collagenase IV STEMCELL, 150 U/ml DNaseI, 0.2 M MgCl_2_, and 0.5 M CaCl_2_). Livers were then incubated in the digestion buffer at 37°C for 45 min while shaking. The digested cell suspension was passed through a 70-μm cell strainer, then centrifuged at 500 × *g* for 7 min at 4°C at least twice, and washed with PBS/2% FCS. To obtain the non-parenchymal fraction, the suspension was centrifuged at 50 × *g* for 4 min, and the supernatant was transferred to a new tube. Non-parenchymal cells were then resuspended in 6 ml of 37% Percoll and centrifuged at 400 × *g* for 10 min (dec.1 acc.9). After a red blood cell lysis step and a further washing step with PBS/2% FCS, cells were used for RNA analysis, FACS sorting at the FACSAria, or phenotypic analysis at the LSR II or Cytek Aurora flow cytometers.

### Kupffer cell isolation

To isolate the Kupffer cells (KCs) out of the total non-parenchymal cell population, liver harvesting and tissue digestion were performed as described above, omitting the red blood cell lysis step. The obtained liver cells were then counted and plated to a concentration of 0.66 × 10^6^/cells in a 12-well plate in cDMEM (10% FCS) for 24 h at 37°C. The KCs were discriminated based on the method of selective adherence, in which only the KCs attach to the plate. The non-adherent cell fraction was removed with gentle PBS washing steps. Functional assays, such as testing efferocytosis efficiency, were performed 48 h after cell plating. Samples where no cells were attached are omitted from the analysis.

### Bile acid measurement

Bile acids were measured using HPLC–ESI–MS/MS. Briefly, samples were prepared using a simple methanol liquid–liquid extraction. Quantitative measurement of bile acids was performed using a LC-ESI-QqQ system run in multiple reaction monitoring (MRM) mode. HPLC analysis was performed using NEXERA X2 LC-30AD HPLC PUMP (Shimadzu) equipped with a Kinetex C18 column (100 Å, 150 mm × 2.1 mm i.d., Phenomenex). For HPLC, a mobile phase A consisting of water and a mobile phase B consisting of acetonitrile methanol (3/1 vol/vol) were used. Both mobile phases were enriched with 0.1% formic acid and 20 mM ammonium acetate. The column was coupled to a QqQ: Q trap 5500 System (SCIEX). Peaks were identified and quantified by comparing retention times, MRM transitions, and peak areas, respectively, to particular corresponding standard chromatograms. Only bile acids that were quantifiable in all samples are depicted in the graphs.

### Patient selection and tissue/blood collection

All patients participating in this study provided written informed consent according to the ethical guidelines of the Institutional Review Board of the medical faculty of the University of Hamburg (PV4081 and PV3548).

### Isolation of PBMCs

Blood from healthy donors and PSC patients, collected in heparin tubes, was diluted 1:2 in PBS. PBMCs were isolated using SepMate tubes (STEMCELL Technologies) according to the manufacturer’s protocol. Briefly, Ficoll-Paque was added to the SepMate tube via the insert, and diluted blood was layered on top. Samples were centrifuged at 1,200 × *g* for 10 min with the brake on. The upper layer was then poured into a new tube and washed twice with PBS/2% FCS. The cells were then stained with fluorophore-conjugated antibodies (see specification below) and phenotypical analysis performed at the Cytek Aurora flow cytometer. The blood withdrawal was approved by the Ethics Committee of Hamburg, and written informed consent was obtained from all patients and healthy controls.

### Isolation of hepatic Mφ from liver biopsy

Liver biopsies from a PSC patient and a non-PSC control patient (healthy liver tissue obtained from a patient with liver adenoma) were collected and transferred into a Petri dish. The tissue was minced into small pieces and digested with 10 ml collagenase buffer (RPMI, 10% FCS, 20 μl 0.5 M CaCl_2_, 10 μl 1 M MgCl_2_, 1 mg DNase, and 1 mg Collagenase) at 37°C for 30 min. Single-cell suspension was obtained by smashing the digestion solution over a 70-μm filter, followed by 30-µm filter. In dependence of the size of the biopsy, multiple washing steps were performed before removing parenchymal cells at 50 × g for 3 min. Afterward, the supernatant was pelleted at 4°C 400 × g for 10 min, and the cells were used for flow cytometry analysis. All steps were performed on ice and low-binding tubes were used coated with MACS buffer for 5 min on ice (DPBS, 1% FCS, and 0.4% 0.5 M EDTA). Antibodies for flow cytometry analysis were purchased from commercial sources (Table 3).

**Table 3.** Human antibody specifications

Epitope	Fluorochrome	Clone	Company	Cat.	RRID
CCR2	PE/Dazzle594	K036C2	BioLegend	357222	AB_2566752
CD14	BUV737	M5E2	BioLegend	301863	AB_2860766
CD16	PerCP-Cy5.5	B73.1	BioLegend	360712	AB_2562955
CD36	BV421	5–271	BioLegend	336230	AB_2814228
CD64	AF700	10-1	BioLegend	305040	AB_2800776
CD66b	PE/Fire640	6/40c	BioLegend	392918	-
CD68	BV785	Y1/82A	BioLegend	333826	AB_2800880
Live/dead	UV-blue	​	TFS	L34962	-
MARCO	PE	PLK-1	eBioscience	12–5447-42	AB_2762430
MERTK	APC	1E+05	R&D	FAB8912A	AB_357213
TIM4	PE-Cy7	9F4	BioLegend	354006	AB_2564542

### Human data analysis

Single-cell/nucleus mRNA sequencing (scRNAseq) data from healthy (*n* = 6), PSC (*n* = 8) and PBC (*n* = 2) human liver samples were obtained from a publicly available dataset (Andrews et al., 2024). Seurat objects were downloaded from the CELLxGENE data portal: https://cellxgene.cziscience.com/collections/0c8a364b-97b5-4cc8-a593-23c38c6f0ac5. Data were analyzed using R (v4.0.5) and the Seurat package (v4.0.4 RRID:SCR_007322). Two distinct gene signatures were derived with the AddModuleScore function. One gene signature was indicative of hepatocyte uptake (containing *ALB*, *CYP3A4*, and *APOA1*), whereas the other one contained genes indicative of phagocytosis (Table S1). Data visualization and statistical analysis were carried out using ggplot2 (v3.3.5 RRID:SCR_014601) and ggsignif (v0.6.4 RRID:SCR_023047).

### Statistics

Statistical analyses were performed using GraphPad Prism (RRID:SCR_002798). Nonparametric tests were used, as the data were not assumed to follow a normal distribution. If a comparison was performed between two unpaired conditions, a two-tailed Mann–Whitney U test was performed. If paired conditions were analyzed, a two-tailed Wilcoxon signed-rank test was applied. Comparisons among more than two related conditions, in which the same cells were exposed to different treatments (within-subject design), were performed using the Friedman test, followed by Dunn’s multiple comparisons test for post hoc analysis. The overall results for the Friedman test are Fig. 4 A P = 0.0007; Fig. 4 D (from left to right) P = 0.0434; P = 0.0342; P = 0.0370; Fig. 4 E P = 0.0009. The results of Dunn’s multiple comparison test are depicted in the figures. Unless otherwise indicated, results were not significant (ns; P > 0.05). Detailed information on the statistical analyses (e.g., group sizes) is provided in each of the figure legends. All data are shown as means ± SEMs; each data point indicates a biological replicate, if not stated otherwise.

The authors used ChatGPT (GPT-5, OpenAI) to assist with English language editing in selected sections of the manuscript. The authors reviewed and take full responsibility for the final content.

### Online supplemental material

Fig. S1 shows the frequency, phagocytic marker expression, and gating strategy of hepatic Mφ from *Mdr2*^*−/−*^ mice and controls at different ages (related to Fig. 1). Fig. S2 shows imaging and flow cytometry data indicating the efficiency of BMDMs and liver Mφ from *Mdr2*^*−/−*^ mice and controls to engulf dying cells in vitro. Fig. S3 shows the involvement of *Lxra/b* in the regulation of cell surface marker expression in BMDMs. Fig. S4 shows the expression of cell surface and intracellular molecules in BMDMs following in vitro uptake of aH, either directly loaded with the bile acid TLCA or in the presence of TLCA in the supernatant (related to Fig. 3). Fig. S5 shows the gating strategy used to analyze efferocytic/scavenger receptor expression in Mφ from the blood and liver of a control and a PSC patient, as well as features of hepatic Mφ analyzed via scRNA-seq (related to Fig. 5). Table S1 shows phagocytic score–associated genes.

## Supplementary Material

Table S1 shows phagocytic score–associated genes.

## Data Availability

The data underlying Fig. 3 B are openly available in the Research Data Repository Zenodo at the link https://zenodo.org/records/20270981.
