# Supplementary material for: Bile acid retention in efferocytic macrophages shapes their inflammatory status during cholangitis
Source: J Exp Med. 2026 Jun 23;223(7):e20242079. doi: 10.1084/jem.20242079 (PMC13289589; doi:10.1084/jem.20242079)
Supplement: Table S1 — shows phagocytic score–associated genes. [file jem_20242079_tables1.pdf]

Table S1. Phagocytic score-associated genes

Gene list - Phagocytic score	
AXL	*GAS6*
MERTK	*PROS1*
STAB1	*THBS2*
STAB2	*MFGE8*
TREM2	*NR1C3 (PPRG)*
CD36	*NR1H3 (LXRA)*
SCARF1	*NR1H2 (LXRB)*
TIMD4	*ABCA1*
ITGB3	*PLTP*
ITGB5	*ABCG5*
BAI1	*SCL2A4*
FCGR1A	*MRC1*
C1QB	*ANXA1*
C4B	*MARCO (SR-A6)*
